# Valorization of Coal Fly Ash into Fe-FAU Catalysts: Targeted Iron Recovery and Fly Ash Matrix Effects in Methane-to-Methanol Conversion

**DOI:** 10.1021/acsomega.6c09000

**Published:** 2026-10-24

**Authors:** Süleyman Şener Akın, Selin Cansu Gölboylu, İlhan Duruk, Can Barkın Dericioğlu, Bahar İpek, Burcu Akata

**Affiliations:** † Micro and Nanotechnology Department, 52984 Middle East Technical University, Ankara 06800, Turkey; ‡ Chemical Engineering Department, Middle East Technical University, Ankara 06800, Turkey; § Central Laboratory, Middle East Technical University, Ankara 06800, Turkey

## Abstract

The valorization of industrial wastes into functional catalysts offers a route toward more sustainable chemical processes, particularly when the inherited complexity of the waste can be retained as a design variable. Here, coal fly ash was converted into Fe-FAU zeolite catalysts through an integrated waste-to-catalyst strategy in which the same industrial residue supplied both the framework-forming Si–Al fraction and the recoverable Fe precursor. During presynthesis acid treatment, Fe was selectively recovered as crystalline Fe­(II) oxalate dihydrate, while the remaining aluminosilicate fraction was transformed into OSDA-free FAU zeolites and subsequently modified by recovered-Fe ion exchange and steaming. Using fly ashes with distinct inherited chemical environments, we show that source-dependent matrix effects govern framework evolution, acidity redistribution, Fe speciation, and catalytic behavior in N_2_O-assisted methane-to-methanol conversion. Isolated Fe^3+^-rich catalysts exhibited the highest initial methanol formation rate of 137 μmol g_cat_^–1^ h^–1^, but showed rapid activity loss associated with accessible acidity and coke formation. In contrast, the Ca-richer Fe-Z2 matrix moderated acid-site accessibility, favored small extra-framework Fe–oxo/hydroxo descriptors, and delivered stable methanol productivity of approximately 71.5 μmol g_cat_^–1^ h^–1^ with suppressed coke formation. Catalysts more susceptible to steaming-induced dealumination or Fe_2_O_3_-like aggregation showed lower sustained methanol productivity. Overall, this work links Fe recovery, OSDA-free FAU synthesis, recovered-Fe incorporation, and MTM catalysis in a single material-flow concept, demonstrating that fly ash acts not as a passive precursor but as a chemically active matrix that controls Fe-site distribution, acidity, and time-on-stream stability.

## Graphical abstract

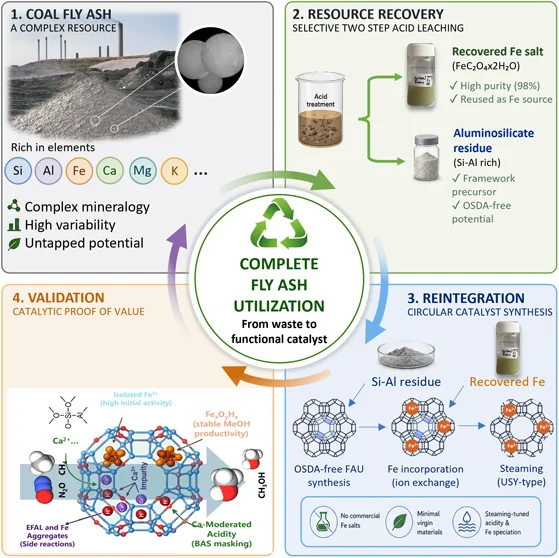

## 1 Introduction

The development of sustainable zeolite synthesis routes is a major challenge in advancing greener chemical technologies. Global demand for zeolites continues to rise due to their broad applications in catalysis, separations, carbon capture, and wastewater treatment. The global zeolite market is projected to exceed USD 6 billion by 2027, yet the cost of synthetic zeolites remains a significant barrier to wider adoption. Among industrial zeolites, FAU-type frameworks hold the largest share, with production volumes reaching several hundred thousand tons per year.^1^ However, conventional FAU synthesis relies on expensive Si/Al precursors. Consequently, fly ash has emerged as an attractive, low-cost raw material for FAU-type zeolites, offering both economic and environmental advantages.^2^

Numerous approaches have been reported for converting fly ash into zeolitic materials, including direct hydrothermal treatment, alkali-fusion-assisted hydrothermal synthesis, microwave-assisted synthesis, and more recently solvent-free or low-solvent routes.^1,3^ Among these, alkali-fusion-assisted hydrothermal synthesis remains one of the most widely used and reproducible strategies for activating the relatively inert glassy Si–Al fraction of fly ash and directing FAU crystallization.^2,4,5^ Despite this potential, most catalytic applications of fly ash-derived zeolites still rely on OSDA-assisted synthesis steps.^6–9^ In several of these routes, fly ash is first converted into a soluble Si–Al-rich fraction, and the separated supernatant is then used as a purified precursor for synthesizing high-silica or OSDA-directed zeolites such as BEA, SSZ-13, or ZSM-5.^7–11^ Although this strategy improves compositional control and broadens framework accessibility, it also decouples zeolite crystallization from the original solid waste matrix and reduces the role of residual cocations and mineralogical inheritance to a precursor-purification issue. Consequently, the compositional complexity of the original fly ash is largely removed rather than incorporated as a functional variable in subsequent zeolite synthesis. FAU-type zeolites offer a distinct advantage for the present integrated strategy because they can be synthesized directly from fly ash without OSDAs while retaining substantial postsynthetic tunability.^12^ Compared with other directly accessible fly ash-derived frameworks such as LTA or CHA, FAU provides a large-pore architecture and more established steaming/dealumination chemistry, enabling acidity, framework stability, and Fe coordination to be adjusted within the waste-to-catalyst pathway. In practice, FAU synthesis from fly ash often involves an acid-washing pretreatment to enhance crystallinity,^13^ yet the leachate generated in this step, frequently rich in Fe, has been largely overlooked as a potential metal precursor. Under appropriate acid selection and leaching conditions, selective Fe recovery becomes feasible, particularly given the inherently high iron content of many fly ash sources. Oxalic acid, a biodegradable chelating agent, selectively complexes Fe^2+^ over Al^3+^ and enables mild extraction of Fe­(II) oxalate dihydrate.^14^ Considering that conventional Fe-zeolite synthesis relies exclusively on commercial Fe salts, the reuse of this recovered Fe represents an unexplored opportunity for circular catalyst design.

For catalytic applications, however, tuning the acidity, porosity, and metal coordination environment typically requires postsynthetic modification of FAU frameworks.^15,16^ Among these treatments, steaming is one of the most widely employed, as it modulates framework Al distribution, acidity, and accessible cation-exchange sites.^17^ Steaming partially redistributes Al and creates coordination environments that remain inaccessible under conventional calcination.^17^ Preparation conditions such as N_2_-protected steaming can regenerate isolated Fe sites via reverse ripening.^18^ Taken together, these preparative steps shape the Al distribution and Fe coordination environments that influence the catalytic performance of the resulting Fe-FAU materials.

One catalytic reaction that is highly affected by the Al distribution and Fe coordination is the direct Methane-to-methanol (MTM) conversion. MTM is both chemically relevant, as methanol is a versatile platform chemical, and also mechanistically diagnostic, due to sensitivity to Fe-mediated redox chemistry, Brønsted acidity (which affects secondary methanol transformation), and hydrothermal framework stability.^19–25^ Zeolites containing redox-active metal cations, particularly Fe, enable the formation of α-oxygen from N_2_O that selectively activates the C–H bond.^23,26–28^ However, methanol selectivity in MTM is strongly influenced by the interplay between Fe speciation and zeolitic acidity.^23^ This interplay is particularly relevant for FAU/Y frameworks, where the large cages and high cation-exchange capacity can accommodate different extra-framework Fe species while acid sites promote secondary methanol transformation. Fe-FAU/Y zeolites have been much less explored for N_2_O-assisted methane oxidation than Fe-MFI, Fe-FER, Fe-MOR, Fe-BEA, Fe-CHA, and Cu-zeolite systems.^19,24,26,27,29,30^ The few Fe-FAU/Y studies available indicate that Fe dispersion, suppression of Fe_2_O_3_ formation, and moderated acidity are critical for improving oxygenate selectivity, making FAU/Y a useful but underexplored framework for probing Fe–acid site interactions in MTM.^31^ Brønsted acid sites (BAS) promote methanol-to-olefin (MTO) and C–C coupling reactions, while Lewis and Extra-Framework Al (EFAL) species redistribute Fe during steaming and alter redox cycling.^20,32–35^ Impurities in fly ash, such as Ca, K, Mg, and native Fe, may therefore play a dual role, although their influence on Fe-zeolite MTM catalysis remains underexplored. Their presence can reshape acidity and modulate the balance between isolated Fe^3+^ species, higher-coordination Fe environments, and Fe–oxo aggregates, each of which may control different aspects of MTM activity and stability.^20,36–39^ This possibility is supported by studies showing that secondary metal cations, such as Zn^2+^ and Cu^2+^, can tune Fe–O intermediates and methanol selectivity in zeolite catalysts. However, in fly ash-derived systems, such impurity-driven effects should not be viewed as isolated promoter or dopant effects.^40–42^ Instead, they arise from the coupled mineralogical and chemical environment inherited from the parent fly ash, where impurity location, glass–crystalline phase distribution, residual cocations, and native Fe speciation collectively define the fly ash matrix effect.^43^

Recent studies have demonstrated the successful conversion of fly ash into catalytically active zeolitic materials, including FAU, ZSM-5, BEA and SSZ-13-type platforms.^10,44–49^ Representative examples are summarized in Table 1. However, fly ash is commonly used primarily as a source of Si and Al, while crystallization is directed by OSDAs, seeds, supplementary silica or alumina sources, and related additives, and catalytically active metals are introduced from external precursors. As reflected in Table 1, inherited species are frequently not considered mechanistically, although some studies relate them indirectly to synthesis or textural properties and a limited number identify specific catalytic functions.^48,49^

**Table 1:** Representative Studies on Fly-Ash-Derived Zeolite Catalysts and the Mechanistic Consideration of Inherited Species from Fly Ash

**reference**	**catalyst**	**fly ash role**	**metal route**	**mechanistic role of inherited species**	**application**
Cocchi et al.^44^	H-FAU	Si–Al precursor	no added metal	none	plastic pyrolysis
Li et al.^45^	BEA	Si–Al precursor	no added metal	none	H_2_O_2_-assisted NO oxidation
Andola et al.^46^	FAU	Si–Al precursor	no added metal	none	glucose-to-HMF
Li et al.^47^	Fe/ZSM-5	Si–Al precursor	external Fe/impregnation	none	H_2_O_2_-assisted NO oxidation
Zhen et al.^10^	Cu–Ce–SSZ-13	Si–Al precursor	external Cu/Ce	none	NO/Hg^0^ removal
Hasan et al.^48^	Ni/Na-FAU	Si–Al precursor	external Ni/impregnation	through texture	CO_2_ methanation
Liu et al.^49^	Cu/Fe–Al–CHA	Si–Al and inherited Fe source	external Cu/one-pot	Cu stabilization	NH_3_–SCR
this work	Fe-FAU	Si–Al and recovered Fe source	recovered Fe/presteaming reintegration	Fe-site distribution and acidity	N_2_O-assisted MTM

Despite these advances, whether the inherited chemistry of fly ash can be retained through zeolite synthesis and post-treatment as an interpretable contributor to catalyst structure and behavior remains insufficiently understood. This question is particularly relevant to fly ash-derived FAU/USY-type catalysts, because crystallization, hydrothermal or steaming-induced framework reorganization, transition-metal redistribution, and acid-site evolution are all sensitive to precursor composition. Accordingly, catalyst behavior cannot necessarily be rationalized from bulk Si, Al, Fe, Ca, or alkali contents alone. Building on the mineralogical perspective of fly ash established by Vassilev and Vassileva,^43^ this work extends the concept toward catalyst design by considering the combined influence of residual cocations, native Fe, recovered Fe, and other source-dependent constituents. These coupled influences are collectively defined here as the fly ash matrix effect.

This study tests the fly ash matrix-effect hypothesis using fly ash-derived Fe-FAU zeolites, with methane-to-methanol conversion serving as a structure-sensitive diagnostic reaction. Three coal fly ashes with distinct chemical and mineralogical characteristics were subjected to the same fractionation–reintegration route. Each ash was separated into an acid-treated aluminosilicate residue and an Fe-rich leachate, which were subsequently reconnected through OSDA-free FAU synthesis, Fe­(II) oxalate recovery, recovered-Fe incorporation, and steaming. In this route, the leachate was converted into a usable Fe precursor, while the solid residue retained source-dependent characteristics relevant to zeolite formation. Incorporation of recovered Fe prior to steaming provided an FAU-specific strategy for examining how inherited chemistry influences Fe redistribution, framework reorganization, acid-site accessibility, and catalytic behavior. A pure FAU-derived reference was included to distinguish these effects from those observed in a compositionally cleaner analogue. Methane-to-methanol conversion was then used to relate source-dependent catalyst chemistry to Fe speciation, acid-site accessibility, product distribution, coke formation, and time-on-stream stability. By reconnecting the aluminosilicate and Fe fractions of fly ash within a common catalyst platform, this work extends the role of fly ash beyond that of a conventional Si–Al precursor toward a chemically integrated resource for zeolite catalyst design.

## 2 Materials and Methods

### 2.1 Materials

In this study, three different local fly ash sources namely Yatağan, Soma and Seyitömer with various impurity profile were selected as silicon, aluminum and iron precursor for Fe-FAU catalyst synthesis to investigate their roles in the methane to methanol reaction. Yatağan fly ash (FA1) was obtained from the Yatağan Termik Power Plant in Turkey. Soma fly ash (FA2) was supplied by the Soma Kolin Power Plant, Seyitömer fly ash (FA3) was obtained from the Çelikler Seyitömer Power Plant in Turkey.

Sodium hydroxide (NaOH) pellets and anhydrous oxalic acid were obtained from Sigma-Aldrich Chemicals. Ammonium chloride (NH_4_Cl) was obtained from Merck Chemicals. Sodium Carbonate (Na_2_CO_3_) was supplied from Kazan Soda Elektrik Company, Turkey. Aluminum Hydroxide powder (Al­(OH)_3_) was supplied by ETI ALUMINYUM. Double-distilled water (DDW) with ≤ 18 MΩ was used in this study. Representative buffered distilled water used as drinking water with pH of 7.3.

### 2.2 Fe­(ii)­oxalate Dihydrate Recovery from Fly Ash Sources

An oxalic acid–H_2_SO_4_ binary acid system, adapted from Yang et al., was used to recover Fe from the fly ash leachates as Fe­(II) oxalate dihydrate. In the present study, this recovery step was integrated with the subsequent OSDA-free FAU synthesis and recovered-Fe incorporation pathway.^14^ In the first step, 8.0 g of fly ash was pretreated with 40 mL of 3 M HCl at 65 °C for 2 h using a liquid-to-solid ratio of 5 mL g^–1^ to remove acid-leachable species. The acid-treated solid was washed with 2 L of deionized water by vacuum filtration and dried. In the second step, 3.0 g of the acid-treated fly ash was mixed with 15 mL of 5 M oxalic acid solution, followed by H_2_SO_4_ addition according to the selected source-dependent acid/fly ash ratios. For FA1 and FA2, 6.75 g of H_2_SO_4_ was used, corresponding to a mass ratio of 1 fly ash:2.25 C_2_H_2_O_4_:2.25 H_2_SO_4_:5 H_2_O. For FA3, 4.5 g of H_2_SO_4_ was used, corresponding to a mass ratio of 1 fly ash:2.25 C_2_H_2_O_4_:1.5 H_2_SO_4_:5 H_2_O. After the second acid-leaching step, the Fe-rich leachate was separated from the solid residue by centrifugation and transferred to a clean bottle using a syringe. The remaining solid residue was washed with 1 L of deionized water and dried, yielding 2.55 g of two-step acid-treated fly ash from 3.0 g of acid-treated fly ash feed for AT-FA1 and AT-FA2 and 2.67 g for AT-FA3, corresponding to approximately 85 wt % and %89 solid yield for the second leaching step, respectively. The pretreated fly ash samples denoted as PT-FA1, PT-FA2 and PT-FA3, while two-step acid-treated fly ash samples were denoted as AT-FA1, AT-FA2, and AT-FA3. The Fe-rich leachate was diluted 2-fold by volume, after which 10.5 g of the diluted leachate was reduced using 100 mg of Fe powder (Fe powder/leachate mass ratio of 1:105) at 50 °C for 1 h. The resulting Fe­(II) oxalate dihydrate salt was recovered by a second centrifugation step. The solution pH values of 0.05 M solutions prepared from salts recovered from all fly ash sources were consistently ∼ 1.9–2.0, in agreement with the Fe^2+^ stability region reported in the literature.^50^

### 2.3 Zeolite Synthesis

FAU-type zeolite synthesis was carried out via alkali fusion-assisted hydrothermal method using ash residues after Fe recovery (AT-FA1, AT-FA2, and AT-FA3).

This method involves high-temperature fusion of the acid-treated fly ash residue with an alkali agent, followed by hydrothermal crystallization of the alkali-fused product in an aqueous medium. Different alkali agents and fusion conditions were selected according to the fly ash source based on our previously published source-specific fly ash zeolite synthesis protocols.^5,51^ Because the ash-derived synthesis mixture was prepared from a heterogeneous alkali-fused solid rather than fully soluble Si and Al precursors, a conventional homogeneous gel composition cannot be defined in the same way as for reagent-based zeolite synthesis.^52^ Therefore, the synthesis composition is reported using operational ratios, including alkali agent/FA mass ratio, solid/liquid ratio, water pH, and added Al­(OH)_3_ where applicable, rather than as a conventional homogeneous gel formula.

For AT-FA1 and AT-FA2, NaOH was used as the alkali agent. The acid-treated fly ash residue was mechanically mixed with NaOH at a AT-FA/NaOH mass ratio of 1:1.2, and the mixture was fused at 750 °C for 1.5 h with a heating rate of 10 °C min-1.^53^ After cooling, the alkali-fused product was ground and used as the solid precursor for hydrothermal synthesis. The fused product was mixed with water adjusted to pH 7.3 at a solid/liquid ratio of 1:3 (w/w), where the solid corresponds to the alkali-fused product and the liquid corresponds to pH-adjusted water. The water pH was selected based on preliminary synthesis optimization to favor FAU crystallization and suppress GIS formation. The suspension was aged under continuous stirring at room temperature for 8 h, followed by crystallization at 85 °C for 15 h. The resulting FAU zeolites were further subjected to a secondary hydrothermal treatment using a zeolite/water ratio of 1:20 (w/w) at 85 °C for 6h. The zeolites obtained from AT-FA1 and AT-FA2 were denoted as Z1 and Z2, respectively.

For AT-FA3, Na_2_CO_3_ was used as the alkali agent. The acid-treated fly ash residue, Na_2_CO_3_, and Al­(OH)_3_ were mechanically mixed at a mass ratio of 2.4:2.76:1 for AT-FA3:Na_2_CO_3_:Al­(OH)_3_, respectively. Al­(OH)_3_ was included in the initial solid mixture to adjust the aluminosilicate composition for FAU crystallization. The mixture was placed in ceramic crucibles and fused at 900 °C for 1 h with a heating rate of 10 °C min^–1^.^51^ The alkali-fused product derived from AT-FA3 was subjected to a standalone hydrothermal synthesis route, in which the fusion product was mixed with deionized water at pH 6.4 and treated under identical aging and crystallization conditions (solid-to-liquid ratio 1:3, 8 h aging at room temperature, 85 °C crystallization for 15 h), without secondary hydrothermal treatment. The resulting zeolite was denoted as Z3.

To isolate the impurity effects, a pure Na-FAU zeolite was synthesized from laboratory-grade chemicals to serve as a reference for comparison. The synthesis procedure can be summarized as follows. In a typical preparation, 12.91 g of Na_2_SiO_3_ was mixed with 43.85 g of deionized (DI) water and stirred for 1 h at room temperature. In parallel, a sodium aluminate solution was prepared by dissolving 1.165 g of NaAlO_2_ in 21.925 g of DI water, also stirred for 1 h at room temperature. While stirring the sodium silicate solution at full speed, the sodium aluminate solution was added dropwise. After an additional 1 h of stirring, a NaOH solution prepared by dissolving 4.595 g of NaOH in 10.96 g of DI water was added to the aluminosilicate gel mixture. This mixture was then aged for 18 h at room temperature. Following the aging step, the gel was subjected to hydrothermal treatment at 95 °C for 24 h to complete the crystallization process. The end product was denoted as PZ.

### 2.4 Fe-FAU Catalyst Design

The postsynthetic treatment sequence was adapted from previous studies on ion-exchanged low-silica FAU stability and steaming of Fe-containing zeolites, but was modified here into a recovered-Fe presteaming incorporation route to enable Fe redistribution and framework reorganization under the influence of the inherited fly ash matrix.^54^ The specific steaming temperature, steam feed, and Fe-loading sequence were adjusted for the fly ash-derived Fe-FAU system investigated here. Accordingly, 1 g of Na-FAU was ion-exchanged with the mixture of 16 mL of 1.5 M NH_4_Cl and 4 mL of 0.05 M Fe­(ii)­oxalate.dihydrate solution and stirred at 90 °C for 1 h to perform Fe ion exchange. After ion exchange, the sample was washed with deionized water and centrifuged three times, followed by overnight drying at 90 °C. The dried NH_4_–Fe–FAU zeolites were then subjected to steaming. The steaming operations were carried out in a Protherm PTF 12/20/250 horizontal tubular furnace as shown in Figure 1.

**Figure 1:**
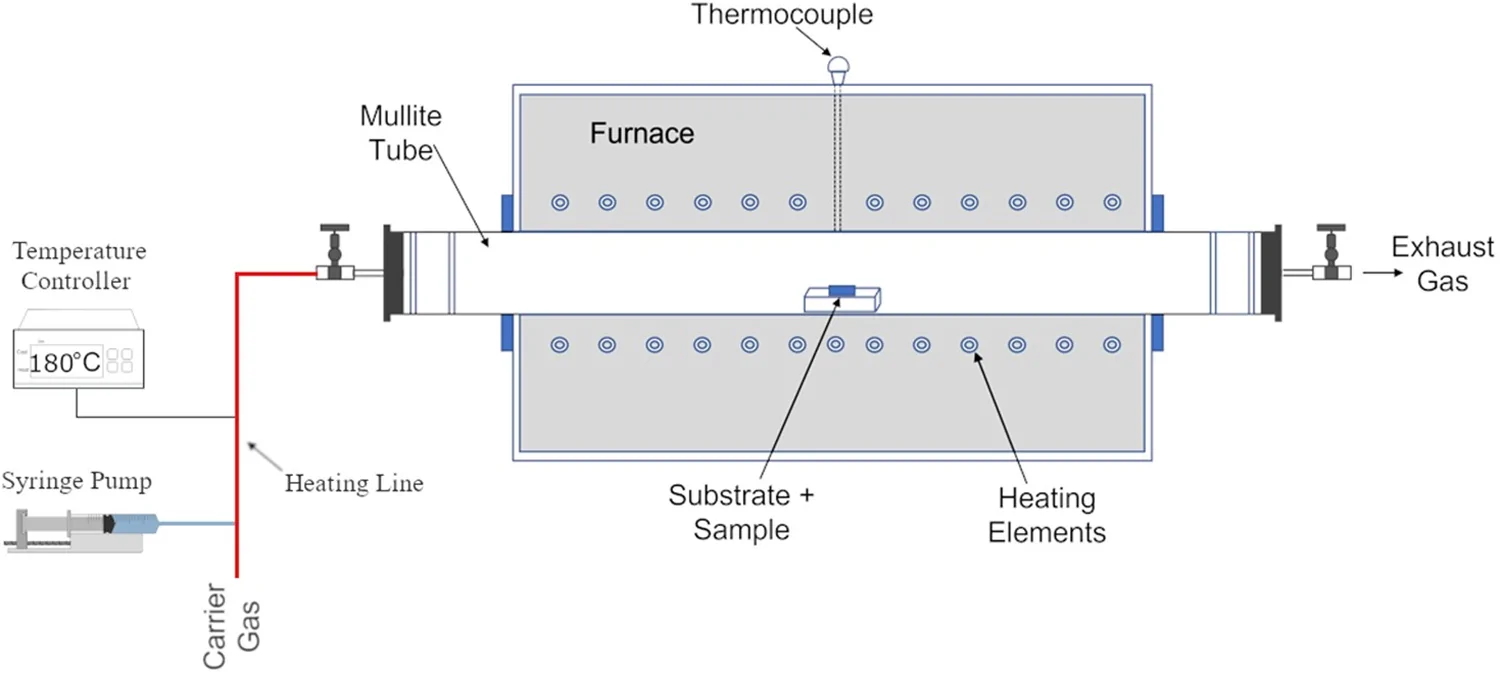
Experimental Setup for Steaming Experiments.

Samples were placed in ceramic crucibles, and the furnace temperature was raised to 600 °C at a heating rate of 5 °C min^–1^ under 10 L min^–1^ N_2_ flow to sweep evolved water and NH_3_. After reaching the target temperature, the samples were exposed to steam for 1 h while maintaining N_2_ as the carrier gas. Superheated steam was generated by passing room-temperature water through a preheated flow line maintained at up to 180 °C and introduced into the system together with nitrogen.^55^ Water was supplied at a flow rate of 1.5 mL min^–1^ using a syringe pump. In this way, controlled humidified N_2_ flow conditions were maintained during the steaming process.

### 2.5 Catalyst Testing

The prepared Fe-containing zeolites were evaluated in the methane-to-methanol reaction under conditions selected with reference to a previous Fe-zeolite MTM study.^26^ Approximately 0.1 g of each Fe-zeolite sample was fixed inside quartz reaction tubes using glass wool. The zeolites were first heated under a helium flow to the reaction temperature (300 °C) at a rate of 5 °C/min. Then, a reaction gas mixture containing %50 He, 30% CH_4_, 10% N_2_O, and 10% H_2_O with a total flow rate of 100 sccm was introduced (60 000 mL/g_cat_/h). The reaction products were continuously and quantitatively analyzed by an online gas chromatograph directly connected to the reactor outlet. A gas chromatography system equipped with both a thermal conductivity detector (TCD) and a flame ionization detector (FID) was used. Methane conversion, methanol selectivity, and methanol production rate per Fe site were calculated using eqs 1–2.

The catalytic test profiles were collected over 10 GC analysis cycles, corresponding to approximately 250 min of time-on-stream. The two GC methods required 21.5 and 27 min of acquisition, respectively, with approximately 4.5 and 4 min required for oven cooling and re-equilibration between successive analyses. The time-on-stream represented by the 10 analytical points was calculated from the nine intervals preceding the final analytical point, comprising six 26 min and three 31 min analysis/cooling intervals [6 × (21.5 + 4.5) + 3 × (27 + 4) = 249 min], reported as approximately 250 min. The cooling period following the final acquisition was not included because no subsequent analytical cycle was performed. The reaction-gas feed was maintained continuously throughout the acquisition and intercycle cooling periods.

1 $$X_{{\mathrm{CH}}_{4}}\left(\%\right)=\frac{r_{{\mathrm{CH}}_{4}}}{F_{{\mathrm{CH}}_{4}}/g_{\mathrm{cat}}}$$

$$S_{{CH}_{3}OH}=\frac{r_{{CH}_{3}OH}}{r_{{CH}_{3}OH}+2{\;}^{*}r_{DME}+r_{\mathrm{CO}}+r_{{\mathrm{CO}}_{2}}}{\;}^{*}100$$ 2

The reaction setup consisted of a quartz tube reactor with an inner diameter of 7 mm and an outer diameter of 9 mm, a furnace controlled by an Ordel OM04 temperature controller, mass flow controllers for helium (carrier gas, 99.999% purity), nitrous oxide (N_2_O, 99%), and methane (CH_4_, 99.995%), a gas wash bottle with adjustable temperature and hence adjustable water partial pressure, 1/8” 316 stainless steel lines with resistive heating bands to maintain 100 °C, and a gas chromatograph (Agilent 7820A) directly connected to the reactor outlet for automatic product sampling as shown in Figure 2.

**Figure 2:**
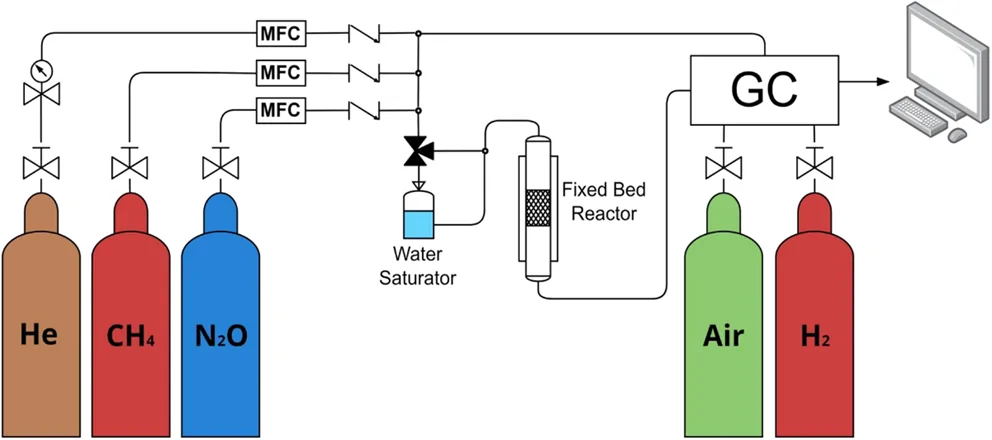
Reaction Setup.

Prior to conducting the control tests, the two columns in the online gas chromatograph PoraPlot Q column (CP7554, 25 m, 0.53 mm, 20 μm) and CP-Molsieve 5Å column (CP7538, 25 m, 0.53 mm, 50 μm) were conditioned at 250 °C for 4 h. Due to this conditioning, calibration tests were repeated 3 times to ensure accuracy.

The regeneration of used catalysts was performed in the same reactor by feeding dry air (Hatgaz, 99.99%) with a flow rate of 75 sccm. The furnace temperature was increased to 550 °C using a heating rate of 5 °C/min and maintained at 550 °C for 2 h. The total CO_2_ amount obtained from the gas chromatograph was used to calculate the total coke amount formed during reaction. The calculated coke rate was included in methane conversion and product selectivity calculations.

Representative repeated synthesis/steaming experiments and short-time MTM repeat tests for Fe-Z2 are provided in the of Supporting Information (See Figures S4, S5 and S6).

### 2.6 Characterizations

Zeolite samples were analyzed with the Rigaku Miniflex XRD instrument in METU Central Laboratory. For the analysis, crystal phases of the samples were determined by scanning with a 0.02 ° step size at a speed of 5 °/min in the range of 5–55 °.

The oxide composition of fly ash samples was determined by the Rigaku ZSX Primus II XRF instrument in METU Central Laboratory. Samples prepared as pellets were scanned in the fluorine–uranium range, and the chemical composition was reported in oxide form.

The chemical composition of catalyst samples was determined by using an Inductively Coupled Plasma-Optical Emission Spectrometer (ICP-OES) in METU Central Laboratory. The instrument used is PerkinElmer Optime 4300DV. The catalysis samples were dissolved in HF/HNO_3_ solution prior to analysis. The Si, Al, Fe, Na, Ca, K, and Mg contents were detected.

The textural properties, specific surface area, pore volume, and average pore diameter of the zeolite samples were assessed by N_2_ adsorption–desorption analyses at 77 K using a Quantachrome Autosorb-6B. The samples were initially degassed with N_2_ at 300 °C for about 3 h to remove residual water and volatiles. The specific surface area was derived based on the multipoint Brunauer–Emmett–Teller (BET) method, whereas micropore volume was determined from t-plot analysis and total pore volume with the Barrett–Joyner–Halenda (BJH) method.

ATR-FTIR spectra were collected using a Bruker IFS 66/S spectrometer equipped with a Hyperion 1000 IR microscope in attenuated total reflectance (ATR) mode. Spectra were recorded over the range of 4000–400 cm^–1^ with a resolution of 4 cm^–1^ and averaged over 32 scans to ensure an adequate signal-to-noise ratio. All spectra were baseline corrected and normalized for comparative analysis.

The framework Si/Al molar ratio was estimated based on the position of the double six-membered ring (D6R) vibration band (ν_3_, typically observed between 550–610 cm^–1^). Due to band splitting in Fe-loaded or steamed samples, the highest wavenumber peak within the ν_3_ region was used for correlation with Si/Al_F_ ratio,^56^ following the empirical relation reported by Salman et al.^57^

3 $$x=3.857-0.00619{\;}^{*}\nu_{3}$$

4 $$\mathrm{Si}/{\mathrm{Al}}_{F}=\left(1-x\right)/x$$

UV–visible absorption spectra of the Fe-containing zeolites were recorded using an Agilent Cary 60 UV–Vis spectrometer. To enhance the signal-to-noise ratio, the samples were prepared by solid-state mixing in a 15:1 ratio of BaSO_4_ to zeolite. Diffuse reflectance data were collected over the 200–800 nm wavelength range. The reflectance data were recorded in reflectance mode and reported as F­(R).

The acidity of the catalysts was measured at the Chemical Engineering Department of İzmir Yüksek Teknoloji Enstitüsü and evaluated by temperature-programmed desorption of ammonia (NH_3_-TPD) using a Micromeritics AutoChem II 2920. Approximately 100 mg of sample was loaded in a quartz microreactor and pretreated in a helium flow (20 mL/min) at 450 °C for 2 h to remove physisorbed species. After cooling to 100 °C, the catalyst was exposed to a 5 vol % NH_3_/He mixture for 45 min to saturate the acid sites. Subsequently, the sample was purged with pure He at 100 °C for 30 min to eliminate weakly adsorbed ammonia and stabilize the baseline. Desorption was carried out by heating from 100 to 700 °C at a linear heating rate of 10 °C/min under a helium flow (20 mL/min). The amount of desorbed NH_3_ was monitored with the TCD signal, which was calibrated using standard NH_3_/He mixtures.

## 3 Results and Discussion

### 3.1 Characterization of Iron Recovery

The chemical compositions of fly ash samples and the left ash residue after iron recovery are shown in Table 2.

**Table 2:** Chemical Composition of Raw and Acid Treated Fly Ash Sources

**sample**	**SiO**_**2**_	**Al**_**2**_ **O**_**3**_	**Fe**_**2**_ **O**_**3**_	**CaO**	**other oxides**
FA1	46.8	25	8.1	11.4	8.7
PT-FA1	58.4	23	8.3	2.3	8
AT-FA1	64.7	22.7	3.8	2.1	6.7
FA2	36.4	23.3	7.8	14.7	17.8
PT-FA2	61.5	21.2	8.3	2.9	6.1
AT-FA2	66.7	20.9	4.7	2.7	5
FA3	50.9	18.5	13.8	4.5	13.2
PT-FA3	63.5	17.7	8.4	1.7	8.7
AT-FA3	68.3	15.6	7.8	1.6	6.7

The selected fly ashes represent distinct mineralogical matrices, where Ca, Fe, Si, Al, and sulfate species may be stored in phases with different acid accessibility, as emphasized by Vassilev and Vassileva.^43^ This is relevant for the Class C sources, since Yatağan fly ash has been reported as a quartz-dominated, low-SO_3_ matrix,^58^ whereas our previous Soma fly ash characterization showed a sulfate-rich composition with anhydrite as a major crystalline phase.^5^ In contrast, Class F fly ash sources are commonly characterized by spherical aluminosilicate domains, where Fe-bearing species may be embedded within quartz- and mullite-rich matrices.^59^

The results indicated that the leachability of calcium and iron was significantly higher for the Class C fly ashes (FA1 and FA2) compared to the Class F sample (FA3). The CaO content decreased from 11.4 and 14.7% to 2.1 and 2.7% for FA1 and FA2, respectively, while in FA3 it decreased only from 4.5 to 1.6%. Similarly, the Fe_2_O_3_ content decreased from 8.1 and 7.8% to 3.8 and 4.7% in FA1 and FA2, whereas in FA3 it decreased from 13.8 to 7.8%. Based on the solid mass balance and XRF-derived Fe_2_O_3_ contents of PT-FA1, PT-FA2, and PT-FA3 and their corresponding residues after the second-stage Fe-targeted leaching treatment, the Fe removal efficiencies were calculated as 61.1, 51.8, and 17.8% for FA1, FA2, and FA3, respectively. These results quantitatively confirm the pronounced source dependence of Fe leachability indicated by the compositional changes.

The limited iron leaching behavior can be attributed to the fly ash matrix characteristics of the different fly ash types. Accordingly, the iron-related minerals are observed predominantly within aluminosilicate microspheres in Class F fly ash sources.^59,60^ This interpretation is consistent with our previous fly ash characterization and zeolite-synthesis studies, where Class F and Class C ashes showed distinct phase-bound Si–Al–Fe–Ca environments and different responses to alkali activation and hydrothermal crystallization.^5,61^ In particular, mullite-rich Class F matrices and Ca-bearing Class C matrices differ not only in bulk oxide composition, but also in the way Fe, Ca, Si, and Al are stored, released, and redistributed during pretreatment.^5,60,62^ The higher Fe leaching efficiency observed for FA1 and FA2 is therefore consistent with the greater acid lability of Ca-bearing phases in Class C ashes.^5,62^ As reported by Lv et.al. (2022), the higher efficiency of iron leaching from FA1 and FA2 might be attributed to the cracked surface during acid leaching, and enhance the diffusion of hydronium ion.^62^ These source-dependent leaching trends indicate that the recoverable Fe fraction is governed not only by bulk Fe content, but also by the mineralogical location of Fe within the fly ash matrix. Moreover, the different leaching responses suggest that matrix-derived differences may persist through pretreatment into subsequent synthesis and post-treatment steps, contributing to the source-dependent Fe-site distribution and acid-site accessibility discussed in the following sections.

Fe recovery route is represented in Figure 3. The XRD pattern of the recovered iron salts obtained from representative Class C and Class F fly ash sources shown in Figure 4a. The XRD patterns confirmed the formation of Fe­(ii)­oxalate dihydrate.^63,64^ Accordingly, leachates yielded crystalline Fe­(II) oxalate dihydrate regardless of the fly ash sources. The corresponding isolated Fe­(II) oxalate amounts were 230, 210, and 63 mg for FA1, FA2, and FA3, respectively. The obtained iron selectivity arises from the lower solubility product of Fe oxalates relative to Ca and Al oxalates.^14^ This was further confirmed by EDX analysis results, as shown in Figure 4b. Together with the absence of other metals in the EDX spectra, these results confirm the successful recovery of Fe­(II) oxalate dihydrate from all fly ash sources.

**Figure 3:**
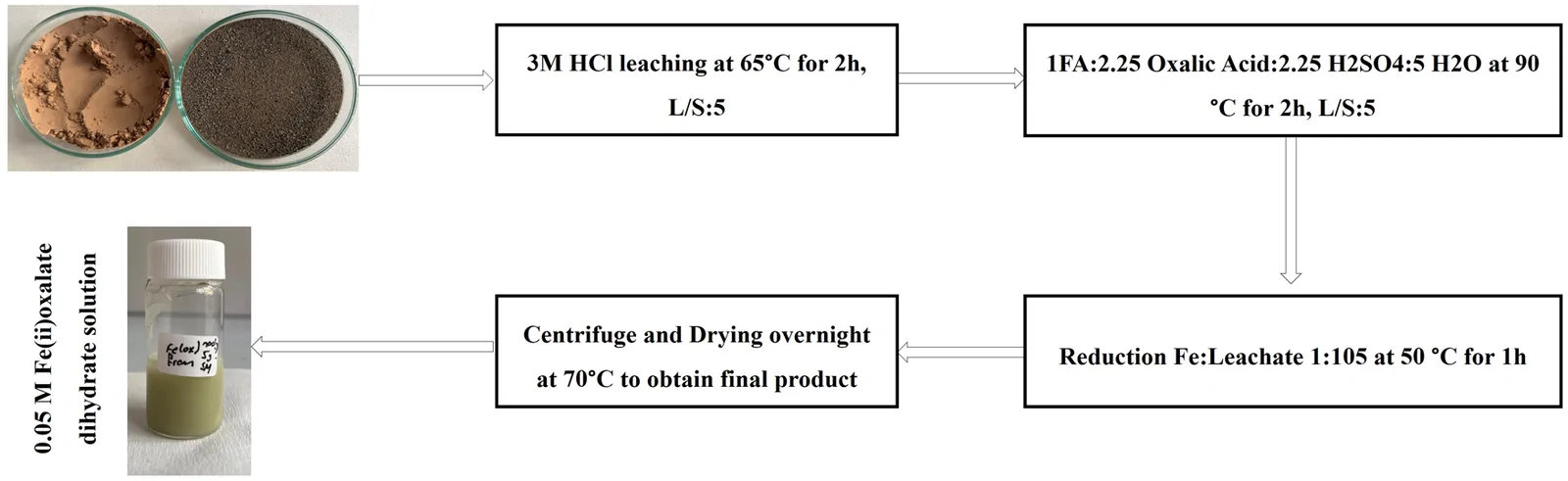
Fe­(ii)­oxalate dihydrate recovery route.

**Figure 4:**
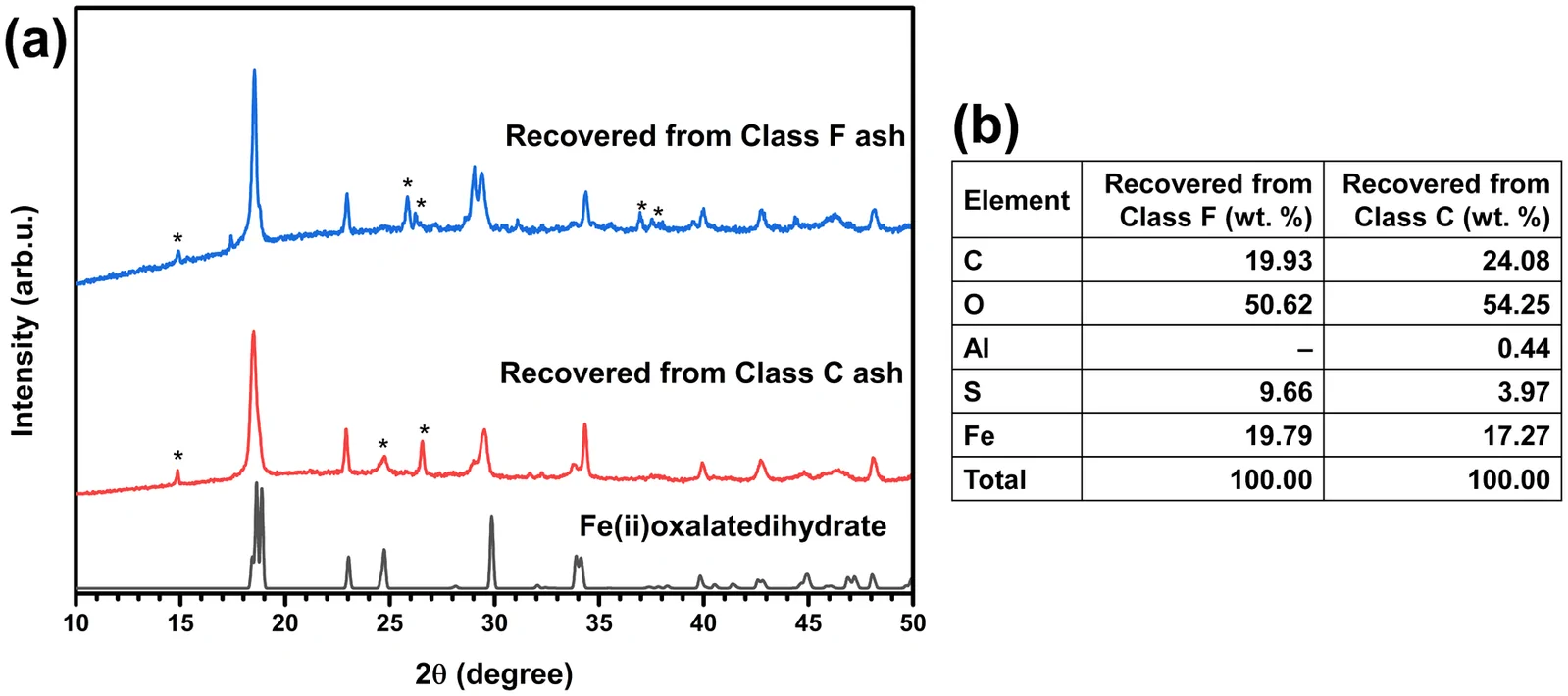
(a) XRD analysis of iron oxalate salt obtained from fly ash sources *: FeSO4.7H_2_O (b) EDX analysis of recovered Fe­(ii)­oxalate dihydrate from Class C and Class F sources.

In line with the oxalate-assisted recovery concept introduced above, the Fe-rich leachate generated during acid treatment was converted into crystalline Fe­(II) oxalate dihydrate rather than being discarded as a secondary acidic waste stream. This step transforms pretreatment from a simple impurity-removal operation into a precursor-recovery stage, linking fly ash purification, Fe recovery, and active-site generation within the integrated Fe-FAU catalyst synthesis pathway.

### 3.2 Characterization of Na-FAU Zeolites

Figure 5 presents the XRD patterns of the fly ash-derived FAU samples obtained using their alkali fused precursors from FA1, FA2 and FA3, along with the FAU zeolite derived from lab-grade chemicals (PZ).

**Figure 5:**
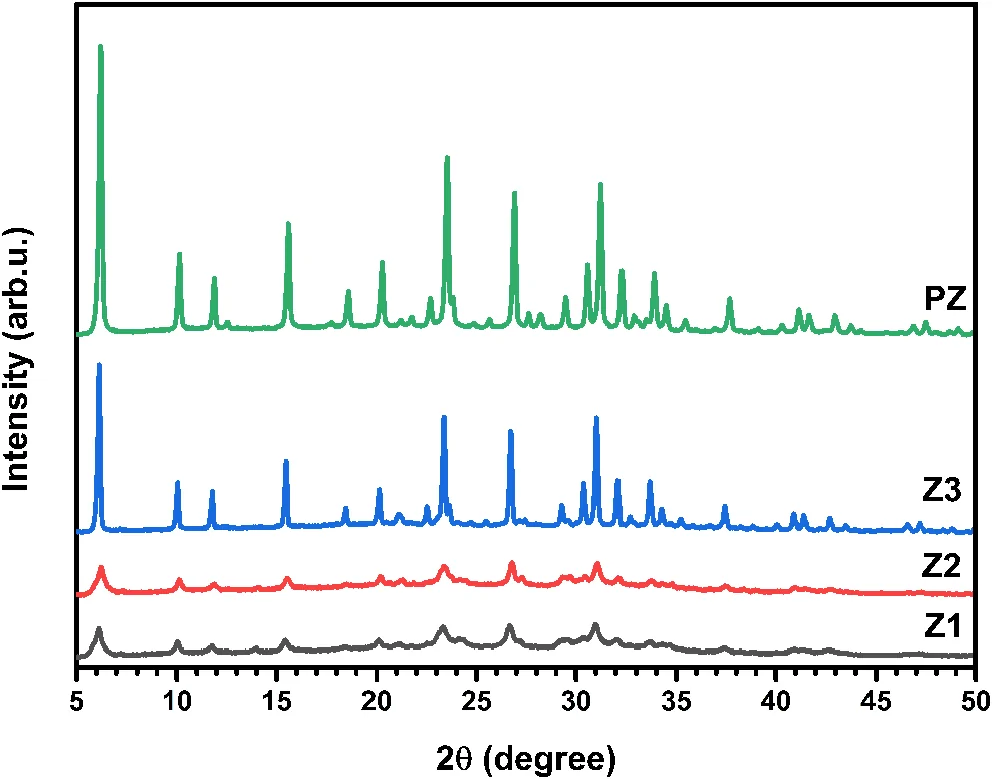
XRD patterns of FAU zeolites of Z1, Z2, Z3 synthesized from AT-FA1, AT- FA2, AT-FA3, and PZ synthesized from lab-grade chemicals.

According to Figure 5, all synthesized zeolites exhibited the characteristic reflections of FAU-type zeolite, confirming FAU as the dominant crystalline phase. Among the fly ash-derived zeolites, Z3 exhibited the highest relative crystallinity, comparable to that of the lab grade chemical-derived FAU reference (PZ), whereas Z1 and Z2 showed lower relative crystallinity. No diagnostic GIS reflections were observed under the optimized synthesis conditions, indicating that the selected hydrothermal conditions favored FAU formation over GIS-type competing phases. FAU crystallization is known to be extremely sensitive to pH and heteroatoms beyond Na, Si, and Al during nucleation and crystal growth.^65^ Thus, the presence of Ca in the products of AT-FA1 and AT-FA2 seemed to suppress long-range FAU ordering.^65–67^ On the other hand, the fusion product of AT-FA3, which retains more Fe after acid washing (Table 2), could suppress the competent phase formation of GIS and evolve toward more ordered FAU-type nuclei under identical conditions.^5^ The present crystallization behavior is consistent with reported trends for multicationic gels in FAU synthesis.^65^ Unlike routes based on purified dissolved Si–Al fractions, the present synthesis follows the acid-treated fly ash solid residue through alkali fusion and hydrothermal crystallization. Thus, residual cocations and mineralogical inheritance remain active variables during FAU nucleation and growth, contributing to the source-dependent crystallinity and textural disorder.

The textural properties of the synthesized Na-FAU zeolites were evaluated by N_2_ adsorption–desorption (BET analysis), and bulk Si/Al ratios were determined by XRF. The corresponding composition and porosity parameters are summarized in Table 3.

**Table 3:** Composition and Textural Properties of Na-FAU Zeolites in This Study

**sample**	**BET surface area** (m^2^ g^–1^)	**total pore volume** (cm^3^ g^–1^)	**micropore volume** (cm^3^ g^–1^)	**average D**_**P**_ **(nm)**	**Si/Al ratio (XRF)**
Z1	379	0.30	0.16	3.2	1.4
Z2	322	0.33	0.10	4.1	1.5
Z3	312	0.23	0.16	2.9	1.2
PZ	653	0.36	0.32	2.2	1.6

The bulk Si/Al ratios of all synthesized zeolites fall within the typical range for FAU-type frameworks.^5,68^ The pure-chemical-derived sample (PZ) exhibited the highest Si/Al ratio of 1.6, while Z3 showed the lowest value of 1.2. Intermediate Si/Al ratios of 1.4 and 1.5 were obtained for Z1 and Z2, respectively. These relatively narrow variations indicate that no substantial differences in bulk Si/Al ratio are present.

BET analysis revealed that the pure-chemical-derived sample (PZ) exhibited the highest surface area (653 m^2^ g^–1^) among all synthesized zeolites. Among the fly ash-derived FAU materials, Z1 showed the highest surface area (379 m^2^ g^–1^), whereas Z2 and Z3 displayed comparable values of 322 and 312 m^2^ g^–1^, respectively. Despite its reduced crystallinity, Z1 maintained a micropore volume (0.16 cm^3^ g^–1^) comparable to Z3. In contrast, Z2 showed the lowest micropore volume (0.10 cm^3^ g^–1^) but the largest mesopore volume (0.23 cm^3^ g^–1^). Moreover, the average mesopore diameters derived from BJH analysis further supported these structural differences due to fly ash matrix effects. PZ and Z3 exhibited the smallest mesopores (2.2 and 2.9 nm), consistent with their higher crystallinity and limited textural disorder. Z1 and Z2 showed a moderately larger average pore size of 3.2 and 4.1 nm, reflecting their partial disorder. These features collectively indicate a more defective framework with larger textural voids for Z1 and Z2, as it aligned with their XRD patterns.

### 3.3 Characterization of Fe-FAU Samples

The crystallographic structure of the Fe-exchanged materials was examined by XRD (Figure 6), while framework vibrations and band shifts associated with Fe incorporation were analyzed by FTIR spectroscopy (Figure 7a–b). The bulk chemical compositions of the Fe-FAU catalysts are summarized in Table 4.

**Figure 6:**
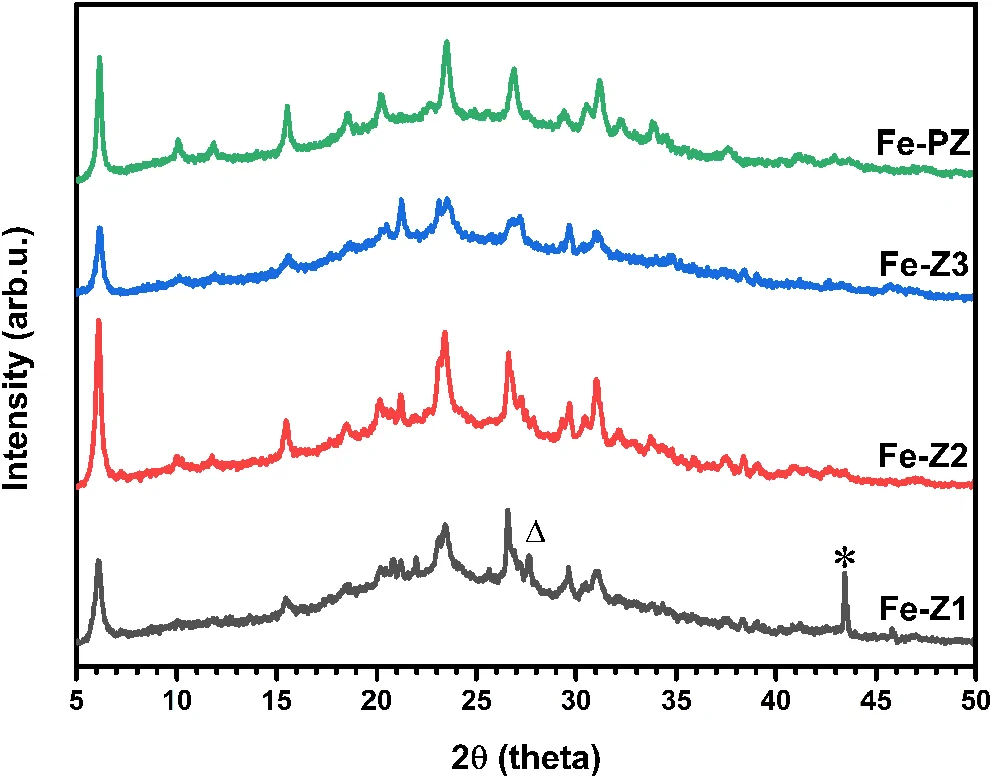
XRD patterns of Fe-FAU samples, Δ: Sillimanite, *: Magnetite.

**Figure 7:**
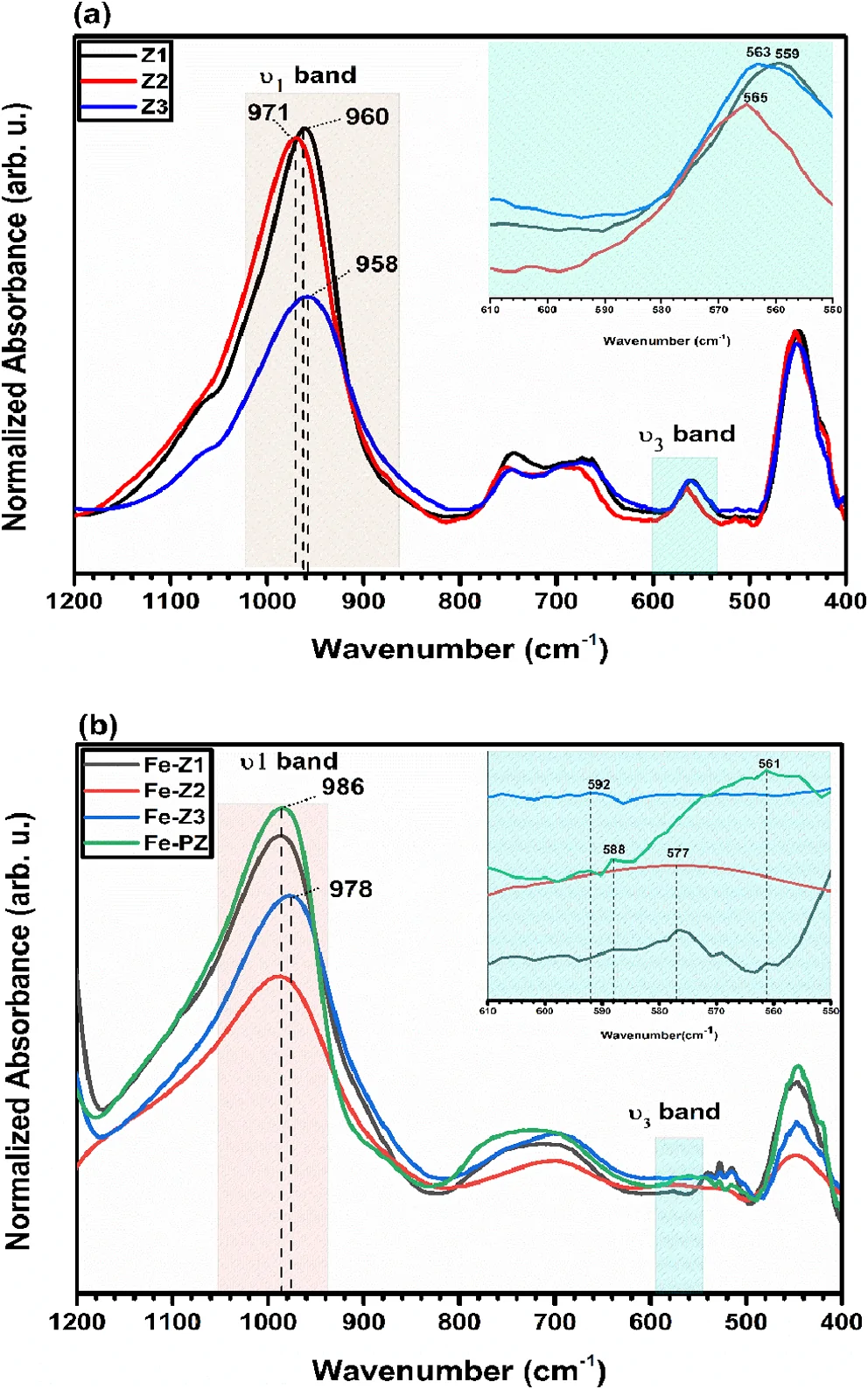
(a) FTIR spectra of synthesized FAU samples from fly ash samples (b) catalyst samples of Fe-Z1, Fe-Z2, Fe-Z3 and Fe-PZ.

**Table 4:** ICP-OES Results of Fe-FAU Zeolites in This Study

**sample**	**Si(%)**	**Al(%)**	**Na(%)**	**Fe(%)**	**Ca(%)**	**K(%)**	**Mg(%)**
Fe-Z1	21	14.5	3.4	3.2	0.9	0.4	0.7
Fe-Z2	20	13.3	2.5	4.6	2	0.4	0.6
Fe-Z3	18.5	15.6	4.3	3.7	0.8	0.3	1.1
Fe-PZ	22	13.7	3.7	1.5			

XRD analysis revealed that Fe-Z2 retained the highest framework crystallinity after hydrothermal steaming compared to Fe-Z1, Fe-Z3, and the pure chemical baseline Fe-PZ. While the presence of extra-framework Fe has been reported to accelerate framework degradation in FAU-type zeolites under severe hydrothermal conditions,^69^ transition metals introduced prior to thermal treatments can counterintuitively mitigate structural collapse in low-silica regimes by stabilizing specific framework sites.^54,70^ The structural resilience observed here demonstrates that the inherited fly ash matrix, acting in tandem with the strategic Fe loading configuration, plays a decisive role in governing long-term hydrothermal stability. The present results indicate that the fly ash matrix has an influence along with proposed Fe loading strategy on structural stability. As determined by ICP–OES and summarized in Table 4, Fe-Z2 exhibits the highest Ca/Al molar ratio of 0.10 and the lowest Na/Al molar ratio of 0.23 among the Fe-FAU catalysts, whereas Fe-Z1 and Fe-Z3 contain lower Ca/Al molar ratios of 0.04 and 0.03, respectively. In contrast, the reference Fe-PZ sample, which lacks stabilizing alkali and alkaline-earth species inherited from fly ash, showed severe crystallinity loss upon steaming. These differences in the final FAU products can be attributed, at least in part, to variations in divalent cation content, since such species strongly influence charge compensation, hydroxyl accessibility, and acid site expression in low-silica FAU frameworks.^35^

The textural and chemical properties of the Fe-FAU catalysts, determined by BET, ICP–OES, and FTIR analyses, are summarized in Table 5. The original N_2_ adsorption–desorption isotherms of the parent and Fe-exchanged catalysts are provided in Figure S2 to support the textural analysis and pore-structure assignment.

**Table 5:** Textural and Chemical Properties of Fe-FAU Catalyst Synthesized from Different Sources

**sample**	**BET surface area** (m^2^ g^–1^)	**total pore volume** (cm^3^ g^–1^)	**micropore volume** (cm^3^ g^–1^)	**Si/Al mol ratio (ICP)**	**Si/Al**_**F**_ **ratio** (FT-IR)	**Fe/Al mol ratio (ICP)**
Fe-Z1	224	0.29	0.06	1.5	2.5	0.11
Fe-Z2	182	0.26	0.05	1.6	2.5	0.17
Fe-Z3	241	0.19	0.06	1.2	4.2	0.12
Fe-PZ	415	0.25	0.16	1.6	3.6	0.05

According to BET results, the Fe-Z1 exhibits the highest mesopore volume, reaching 0.23 cm^3^ g^–1^, followed by Fe-Z2 with 0.21 cm^3^ g^–1^. In contrast, Fe-Z3 and Fe-PZ can be classified as low-mesoporosity samples, with mesopore volumes of 0.13 and 0.09 cm^3^ g^–1^, respectively, and Fe-Z3 displaying the lowest total pore volume. The loss of microporosity follows the crystallinity trend observed by XRD. Fe-Z2 retained approximately 50% of its original micropore volume after steaming, whereas Fe-Z1 and Fe-Z3 experienced more pronounced losses of about 63%. The pronounced micropore collapse in Fe-Z3 may be associated with the formation of iron oxide aggregates or nonframework Al species that partially block the FAU pore system, as previously reported for steamed or metal-modified zeolites.^71^

The FTIR band positions and framework Si/Al_F_ ratios of the Fe-FAU samples derived from infrared analysis are summarized in Figure 7a,b and Table 5.

In particular, the ν_3_ double-ring vibration bands of Fe-Z1 and Fe-Z3 exhibit pronounced blue shifts, moving from 559 and 563 cm^–1^ to 577 and 592 cm^–1^, respectively. In contrast, Fe-Z2 shows a less pronounced shift within the ν_3_ band region from 565 to 577 cm^–1^. In addition, the ν_1_ asymmetric T–O–T stretching vibrations shifted from 960, 971, and 959 cm^–1^ in the parent samples to 986, 986, and 978 cm^–1^ for Fe-Z1, Fe-Z2, and Fe-Z3, respectively. These spectral changes confirm that steaming induces partial dealumination, as evidenced by the blue shifts in the ν_3_ and ν_1_ bands, which are widely recognized diagnostic markers for framework Al removal in FAU-type zeolites.^56,72^ Thus, these shifts correspond to the formation of various nonframework Al species, consistent with the FTIR signatures reported for steamed or metal-modified USY.^56,72,73^ Notably, Fe-Z2 exhibited less pronounced ν_3_ shifts and no observable band splitting compared to the other samples, suggesting a lower extent of framework rearrangement under identical steaming conditions. Considering the higher Ca/Al molar ratio of Fe-Z2, this behavior may reflect matrix-assisted modulation of the dealumination/reorganization pathway rather than complete framework stabilization. These FTIR observations are in good agreement with the XRD and BET results, which likewise indicate a less structural alteration in the Fe-Z2 sample. The results indicate that the fly ash matrix exerts a significant influence on the dealumination behavior of fly ash-derived Fe-FAU samples.

Beyond framework rearrangement, steaming also governs the distribution and accessibility of zeolitic acid sites by converting ammonium species to Brønsted protons and inducing partial dealumination, while the presence of alkaline earth cations such as Ca^2+^ can moderate this process through charge compensation and site masking. The hydroxyl stretching region and Lewis acid site-related framework perturbation region of FTIR spectra of the Fe-FAU samples are depicted in Figure 8.

**Figure 8:**
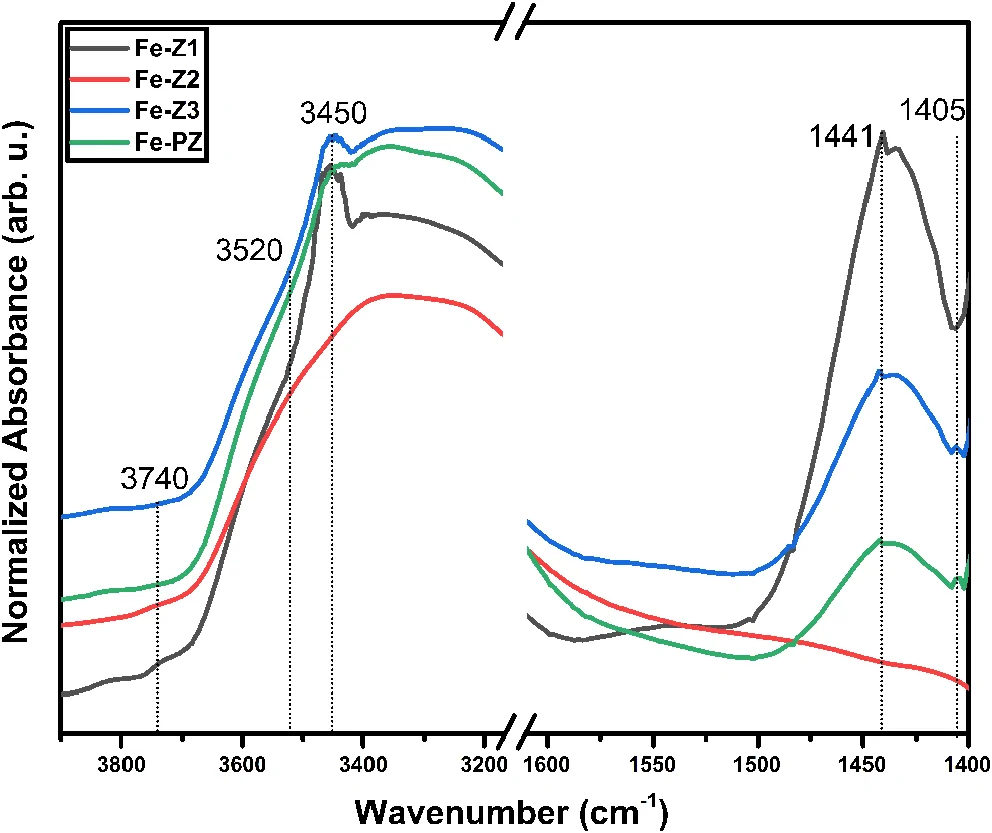
FTIR spectra in the OH stretching vibration region (3800–3400 cm^–1^), and Lewis-acid-related framework perturbation region (1400–1600 cm^–1^) of Fe-FAU samples.

Fe-Z1 displays multiple resolved features within 3400–3800 cm^–1^, including shoulders near 3740 and 3520 cm^–1^ and a structured band in the 3430–3480 cm^–1^ range, consistent with accessible Brønsted hydroxyls and perturbed OH groups associated with framework Al.^56,72^ Fe-Z3 and Fe-PZ exhibit similar but weaker features, indicating a lower population or accessibility of such hydroxyl species. The samples specifically showed distinct characteristics at 3450 cm^–1^, which is hydroxyl bonds located at S_II_ sites in FAU cages.^56^ In that regard, Fe-Z2 shows a markedly suppressed OH stretching signal, suggesting that a substantial fraction of framework-associated hydroxyls are either charge-compensated or spectroscopically masked by Ca^2+^ species introduced during synthesis and retained after steaming.^34,35,74^

Complementary trends are observed in the 1400–1600 cm^–1^ region as represented in Figure 8, which is commonly associated with lattice vibrations sensitive to accessible Lewis-type environments and extra-framework aluminosilicate species, even in the absence of probe molecules. Fe-Z1, Fe-Z3, and Fe-PZ exhibit a distinct band near 1440 cm^–1^, typically attributed to extra-framework Al species or nonframework aluminosilicate environments. An additional shoulder near 1405 cm^–1^ is observed for Fe-Z3 and Fe-PZ, consistent with a higher contribution from Lewis-acid-like environments formed during steaming.^35,74^ Notably, Fe-Z2 lacks a distinct feature in this region, indicating a reduced contribution from such accessible Lewis acidic environments, in agreement with its higher Ca/Al molar ratio (Table 4).

Taken together, the FTIR results indicate that Fe-Z2 undergoes selective masking and accessibility reduction of both Brønsted hydroxyls and Lewis-type environments, whereas Fe-Z1, Fe-Z3, and Fe-PZ retain more spectroscopically visible hydroxyl and nonframework aluminosilicate features. These differences reflect acidity redistribution and accessibility modulation, rather than simple changes in total acid site density.

To quantitatively resolve how these spectroscopic differences translate into acidity strength distribution and accessibility, NH_3_-TPD analysis was performed and calculated acid site amounts represented in Table 6 and deconvoluted figures shown in Figure S3. The raw NH_3_-TPD profiles and deconvoluted curves are provided in Figure S3, while the corresponding fitting parameters, including peak positions, integrated areas, and calculated NH_3_ amounts for each desorption domain, are summarized in Table S1.

**Table 6:** Amount of Weak, Medium and Strong Acid Sites, from the NH_3_-TPD Data

	**acid sites by NH3-TPD** (mmol/g)				
**sample**	**weak**	**medium**	**strong**	**very strong**	**total**
Fe-Z1	0.16	0.34	0.23	0.07	0.81
Fe-Z2	0.16	0.36	0.17	0.07	0.76
Fe-Z3	0.11	0.26	0.05	0.05	0.47
Fe-PZ	0.16	0.30	0.15	0.03	0.64

The NH_3_-TPD profiles were deconvoluted into fitted desorption components and evaluated according to their peak centers, integrated areas, and calculated NH_3_ amounts. Following literature reports on zeolitic acid-site analysis, the fitted components were assigned to weak (100–250 °C), medium (250–350 °C), strong (350–500 °C), and very strong (500–650 °C) acidity domains.^68,75–77^ The very strong domain is interpreted as a high-temperature NH_3_ desorption contribution from strongly retained NH_3_ on acid-related, extra-framework, and/or Fe-containing environments.^68,75–77^

NH_3_-TPD deconvolution shows that in the weak acidity domain (100–250 °C), Fe-Z1, Fe-Z2, and Fe-PZ exhibit comparable contributions (0.16 mmol g^–1^ each), while Fe-Z3 shows a lower fraction (0.11 mmol g^–1^), indicating an overall suppression of accessible acidity in Fe-Z3. In the medium acidity domain (250–350 °C), Fe-Z2 exhibits the highest contribution (0.36 mmol g^–1^), slightly exceeding Fe-Z1 (0.34 mmol g^–1^), Fe-PZ (0.30 mmol g^–1^), and Fe-Z3 (0.26 mmol g^–1^), indicating redistribution toward moderately bound, structurally coupled acid sites.

In contrast, the strong acidity domain (350–500 °C) shows a clear suppression in Fe-Z2 (0.17 mmol g^–1^) relative to Fe-Z1 (0.23 mmol g^–1^), while Fe-PZ (0.15 mmol g^–1^) and Fe-Z3 (0.05 mmol g^–1^) exhibit progressively lower contributions. Notably, this selective reduction of strong acidity in Fe-Z2 provides direct quantitative evidence for acidity moderation at comparable Si/Al_F_ to Fe-Z1, indicating that highly reactive, dehydration-prone acid sites are suppressed without extensive framework dealumination. The very strong acidity domain (500–650 °C), attributed to strongly bound ammonia on extra-framework aluminosilicate species, framework–extra-framework Brønsted environments, and strongly coordinated Fe clusters.^75^ This is present in Fe-Z1 and Fe-Z2 (0.07 mmol g^–1^ each), but reduced in Fe-PZ (0.03 mmol g^–1^) and Fe-Z3 (0.05 mmol g^–1^).

Overall, total NH_3_ uptake follows the order Fe-Z1 (0.81) > Fe-Z2 (0.76) > Fe-PZ (0.64) > Fe-Z3 (0.47 mmol g^–1^). The relatively high total NH_3_ uptake of Fe-Z2 compared with Fe-PZ and Fe-Z3 is consistent with its lower Si/Al_F_ ratio, however, compared with Fe-Z1, Fe-Z2 shows a lower contribution in the 350–500 °C desorption domain and a higher contribution in the 250–350 °C domain. This indicates that Fe-Z2 does not simply undergo acidity loss, but rather exhibits Ca-associated modulation of acid-site accessibility and NH_3_-binding environments.^34,72,78^ Fe-Z1 and Fe-Z2 exhibit comparable framework Si/Al_F_ values after steaming, approximately 2.5, as supported by their double-ring vibration positions. Therefore, the difference in acidity behavior cannot be attributed simply to different degrees of framework dealumination. Under this comparable framework composition, Fe-Z2 shows suppressed OH stretching features, reduced extra-framework/Lewis-type FTIR bands, and redistribution of NH_3_ desorption from the 350–500 °C domain toward the 250–350 °C domain. Together with the higher residual Ca/Al ratio of Fe-Z2 and literature reports on alkaline-earth-metal moderation of zeolitic acidity, these results suggest Ca-associated modulation of acid-site accessibility and NH_3_-binding environments.^35,79^

Taken together, the combined ICP, FTIR, and NH_3_-TPD results indicate that Ca^2+^ plays a moderating role on zeolitic acidity in Fe-Z2, selectively suppressing both Brønsted hydroxyl accessibility and the formation of exposed Lewis-acid-type environments without inducing severe framework disruption. This redistribution of acidity provides a mechanistic basis for the differences in catalytic behavior observed during MTM conversion.

### 3.4 Structure–Activity Relationship of Fe-FAU catalyst in MTM reaction

The characterization results demonstrated that each Fe-FAU catalyst retained distinct structural, acidic, and electronic features inherited from its parent fly ash source. These differences were reflected in framework stability after steaming, the strength of acid sites, and the coordination environment of Fe species. To understand how these characteristics influence the methane-to-methanol reaction, UV–Vis spectroscopy and NH_3_-TPD were evaluated alongside catalytic performance.

The Fe sites are detected by UV–VIS spectroscopy and represented in Figure 9a. The UV–Vis features were assigned to (i) isolated extra-framework Fe^3+^ species at ∼240–250 nm, (ii) higher-coordination extra-framework Fe_*x*_O_*y*_H_*z*_ species at ∼290–320 nm, and (iii) larger Fe oxide/Fe_2_O_3_-like aggregates above 320 nm.^77,80^ Here, Fe_*x*_O_*y*_H_*z*_ denotes small extra-framework Fe–oxo/hydroxo environments inferred from the UV–Vis response, and is used as a comparative descriptor rather than as definitive evidence for a uniquely defined Fe nuclearity or active-site geometry. NH_3_-TPD complements this by probing acid site characteristics, which control methanol dehydration, olefin formation, and coke pathways.^76,81^ Taken together, UV–Vis and NH_3_-TPD provide coupled descriptors for correlating Fe-site distribution, acidity, and catalytic behavior under MTM conditions.

**Figure 9:**
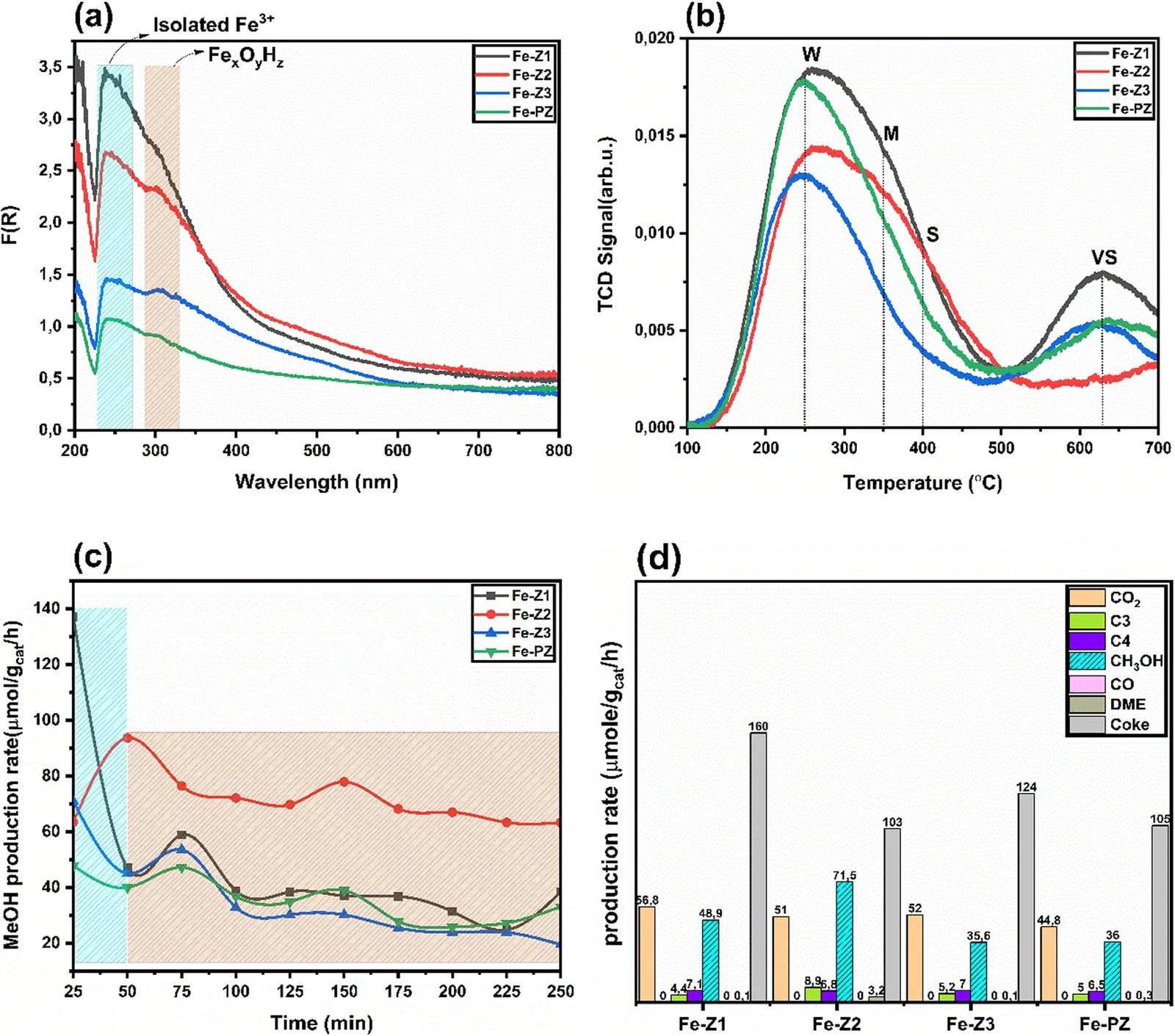
(a) UV–Vis spectra of Fe-FAU samples (b) NH3-TPD analysis of Fe-FAU samples, W­(weak), M­(medium),S­(strong) and VS (very strong) acid sites (c) Methanol production trend for a 4 h period (d) average production rates.

UV–Vis results indicated that the Fe incorporation method used in this study generated a distribution of catalytically relevant Fe environments, including isolated extra-framework Fe^3+^ descriptors together with oligomeric and more highly coordinated Fe environments.^77^ Accordingly, two primary UV–Vis features were resolved for the catalyst series. The absorption band at ∼240–250 nm is assigned to isolated extra-framework Fe^3+^ species located at charge-exchanging sites, whereas the broader feature extending over ∼285–320 nm can comprise several overlapping extra-framework Fe environments, including dimeric Fe species, small Fe-oxo/Fe-oxo-hydroxo clusters, and precursors to larger aggregates.^24,26,38,80^ Because these closely related contributions cannot be unambiguously distinguished by UV–Vis spectroscopy alone, they are collectively denoted here as small Fe_*x*_O_*y*_H_*z*_ species rather than assigned to a single discrete Fe structure. This distinction is catalytically relevant, as dimeric and small oligomeric Fe-oxo environments may provide redox-active configurations favorable for selective methane oxidation, whereas progressive Fe aggregation can promote less selective reaction pathways. Nonetheless, the spectra indicate that the parent fly ash matrix appears to influence the structural evolution and relative distribution of these diverse Fe environments. Parallel to the severe framework degradation observed via XRD and the pronounced structural band shifts in FTIR for the Class F-derived Fe-Z3 sample, distinct spectral shoulders emerged near 320 and 500 nm. These features, highlighted in the deconvoluted spectra (Figure S1), are consistent with bands above 350 nm commonly associated with Fe_2_O_3_-like aggregates, implying partial iron agglomeration driven by residual iron oxides inherited from the parent byproduct.

Moreover, the two Class C-derived samples revealed contrasting coordination preferences. Fe-Z1 exhibited a superior signal intensity at ∼240–250 nm, indicating a larger population of isolated extra-framework Fe^3+^ sites, while Fe-Z2 displayed a more pronounced contribution within the ∼285–320 nm region associated with oligomeric Fe_*x*_O_*y*_H_*z*_ environments.^24^ Within this broader population, contributions from dimeric and small Fe-oxo/Fe-oxo-hydroxo species may be particularly relevant to the comparatively sustained methanol productivity of Fe-Z2,^20,36^ although the individual species cannot be uniquely distinguished by UV–Vis spectroscopy alone. Because cocations can significantly perturb the local electronic environment and coordination accessibility of transition metals within zeolitic channels,^76^ the subtle differences in active site topology between Fe-Z1 and Fe-Z2 are likely influenced by their distinct alkali and alkaline-earth cation profiles, which reflect both the measured residue composition and the parent matrix differences discussed above. Specifically, the inherited matrix contributes to divergent Fe site redistributions during steaming despite the nearly identical bulk chemical compositions of the ash residues remaining after initial iron recovery (e.g., AT-FA1 and AT-FA2, Table 1). Conversely, the pure chemical reference, Fe-PZ, displayed the lowest iron incorporation due to the absence of native, pre-existing iron species, suggesting that structural iron remnants within the waste matrix influence subsequent Fe incorporation pathways.

The catalytic performance of these Fe-FAU samples in direct methane-to-methanol (MTM) conversion was evaluated using N_2_O as an oxidant, serving as an uncompromising, structure-sensitive chemical probe of the redox nature and stability of the waste-recovered active sites (Figure 9c,d). To fully map these pathways, coke-excluded, time-resolved product selectivity profiles are provided in Figure S5, illustrating the methanol-preserving behavior of Fe-Z2 against the more pronounced CO_2_/C_3_–C_4_ hydrocarbon enrichment observed over the other catalysts.

Clear variations in activity, selectivity, and stability were observed across the catalyst series, showing that identical synthetic protocols can yield markedly different catalytic outcomes depending on the source byproduct matrix. The Class C-derived Fe-Z1 and Fe-Z2 catalysts achieved substantially higher average methanol production rates (48.9 and 71.5 μmol g_cat_^–1^ h^–1^, respectively) than either the Class F-derived Fe-Z3 or the pure chemical Fe-PZ baseline, both of which plateaued at approximately 35.6 μmol g_cat_^–1^ h^–1^. However, their time-on-stream stability profiles diverged sharply. While Fe-Z1 reached the highest initial methanol formation rate of 137 μmol g_cat_^–1^ h^–1^, it underwent rapid, severe activation loss. In contrast, Fe-Z2 maintained a remarkably stable productivity profile at 71.5 μmol g_cat_^–1^ h^–1^ throughout the testing window. Fe-Z3 exhibited a transient initial rate of 72 μmol g_cat_^–1^ h^–1^ before decaying to 19 μmol g_cat_^–1^ h^–1^, indicating pronounced time-on-stream deactivation. Its Fe_2_O_3_-like UV–Vis features suggest that a larger fraction of Fe was present in aggregated environments, which may restrict the population of accessible redox-active Fe sites available for sustained catalytic turnover. The consistently lower performance of the synthetic Fe-PZ reference is mainly associated with its restricted iron loading (Fe/Al = 0.05, Table 5), is consistent with a lower abundance of potentially accessible Fe redox sites.

These kinetic profiles correlate with the underlying spectroscopic site distributions. The high initial methanol productivity of Fe-Z1 might be attributed to its larger contribution of isolated Fe^3+^ descriptors, since such mononuclear Fe environments are well established in Fe-zeolite/N_2_O chemistry as precursors that form reactive high-spin Fe­(IV)=O species upon N_2_O activation, enabling cleavage of the strong methane C–H bond.^27^ However, its rapid decay indicates that isolated Fe-rich environments can promote high initial methanol productivity, but require sufficient active-site longevity to sustain MTM performance over time-on-stream.^32,36,39,80^ Conversely, the sustained productivity of Fe-Z2 corresponds to its prominent extra-framework Fe_*x*_O_*y*_H_*z*_ features. Considering the contribution in the ∼ 285–320 nm region together with the catalytic response, small multinuclear Fe-oxo/hydroxo species are likely to constitute a catalytically relevant fraction of this population. In particular, dimeric configurations such as [Fe_2_O_2_]^2+^ and [HO–Fe–O–Fe–OH]^2+^, proposed in literature DFT models to participate in methane activation and redox cycling, represent plausible structural motifs within this broader Fe_*x*_O_*y*_H_*z*_ population.^20,26,36^ However, as these individual configurations cannot be uniquely resolved by UV–Vis spectroscopy, Fe_*x*_O_*y*_H_*z*_ is retained as a collective descriptor for multicenter Fe-oxo/hydroxo environments that may provide comparatively persistent redox-active configurations under the present reaction conditions.

Ultimately, the stable productivity of Fe-Z2 is consistent with a favorable redox–acid balance. The prominent contribution of these extra-framework Fe_*x*_O_*y*_H_*z*_ environments coincides with moderated acid-site accessibility, consistent with the maintenance of oxidation capacity while suppressing dehydration-driven coking pathways.^36,80,82–84^ The importance of this coupling is further supported by recent Fe-zeolite studies demonstrating that framework Al arrangement regulates Fe-site location and speciation while the associated proton environments influence subsequent methanol-to-hydrocarbon chemistry. Thus, the Fe environment responsible for primary methane oxidation and the acid-site environment governing secondary methanol conversion cannot be considered independently when interpreting the observed selectivity and stability.^77^ Nonetheless, these site-dependent trends must be evaluated within the strict framework and protocol constraints inherent to Fe-zeolite MTM chemistry. Because methanol productivity and selectivity are strongly governed by topology, Si/Al ratio, feed composition, and activation protocols, empirical performance rankings against optimized high-silica commercial systems like Fe-CHA, Fe-MOR, or Fe-MFI are methodologically inappropriate.^24,26,31,80,85^ The fly ash-derived Fe-FAU catalysts synthesized herein operate in a substantially lower Si/Al regime even after steaming, placing them among the most aluminum-rich Fe-zeolite platforms investigated for N_2_O-assisted MTM. While this low Si/Al matrix provides a highly acid-dense environment that can facilitate acid-catalyzed methanol retention, dehydration, and subsequent coke deposition, the inherited cocation environment can contribute to regulating the accessible redox-site population for N_2_O activation. Consequently, this work should be contextualized not by absolute productivity benchmarking against optimized synthetic model catalysts, but by the mechanistic insight it provides into selectivity evolution and active-site regulation within an integrated, low-Si/Al, matrix-derived Fe-FAU platform.

This low-Si/Al framework environment clearly influences on the secondary product distribution. Because all samples remain within a highly acid-dense regime, framework acidity influences the competitive balance between selective methanol survival and secondary dehydration side reactions. While the chemical reference Fe-PZ possesses a lower total acidity than Fe-Z1 or Fe-Z2, its product profile cannot be interpreted solely via total NH_3_ uptake. Due to its limited iron loading, Fe-PZ suffers from restricted primary methanol formation. However, it retains a sufficiently high enough acid-site density to promote dehydration of the trace methanol produced, causing light olefins to appear prominently in its product stream. Thus, the olefin-to-methanol ratio appears to reflect the localized balance between Fe redox-site density and acid-site accessibility, rather than bulk acidity totals. This is supported by carbon-selectivity analysis and time on stream full product distributions (Table S2 and Figure S5), where the impurity-free Fe-PZ reference displayed identical carbon selectivities for both olefins and methanol (15.9%), while yielding a moderate coke selectivity (46.5%) and suppressed overoxidation (19.8% CO_2_ selectivity). This behavior is consistent with its higher framework Si/Al_F_ (3.6) and a lower total acid density (0.60 mmol NH_3_/g), allowing lighter olefinic intermediates to escape without extensive chain-growth into heavy coke components.^78,86^

In contrast, Fe-Z1 exhibited a lower olefin-to-methanol ratio but suffered from the highest coke deposition rate (160 μmol g_cat_^–1^ h^–1^), consistent with its high total acidity (0.81 mmol NH_3_ g^–1^) and extensive secondary conversion of methanol-derived intermediates. Such behavior is consistent with experimental studies of continuous CH_4_/N_2_O oxidation over Fe-zeolites, where retained methanol/methoxy species were associated with subsequent carbon formation and increasing coke accumulation was accompanied by progressive loss of catalytic activity. Chow et al. further identified coke as a secondary reaction product and showed that conditions facilitating methanol removal suppressed its formation, whereas Zhao et al. correlated the strongest time-on-stream decay among Fe-ZSM-5, Fe-β, and Fe-FER with the greatest coke accumulation.^80,82^ Accordingly, coke formation in the present catalysts is considered not only a competing carbon sink but also a plausible contributor to the progressive restriction of accessible catalytic sites. Quantitatively, carbon-selectivity data is consistent with coke as the dominant carbon sink for Fe-Z1 (52% C-selectivity), supported by intense FTIR hydroxyl fingerprints in the 3400–3800 cm^–1^ region consistent with a comparatively abundant hydroxyl population. Crucially, the attenuated FTIR signatures observed for Fe-Z2 across both the hydroxyl (3400–3800 cm^–1^) and intermediate (1400–1600 cm^–1^) framework vibrations coincide with its suppressed olefin and coke production. The slightly higher residual Ca and Mg content within the Fe-Z2 matrix may contribute to the moderation of acid-site accessibility, as evidenced by the redistribution of NH_3_ binding sites from high-temperature to intermediate-temperature desorption domains relative to Fe-Z1 (Table 6).^35,78,87^ This matrix-associated moderation is consistent with its more favorable selectivity profile, where Fe-Z2 yielded the lowest coke carbon selectivity (37.5%) and the highest overall methanol selectivity (25%) among all tested materials, allowing it to achieve a stable average productivity of 71.5 μmol g_cat_^–1^ h^–1^.

Finally, the performance of Fe-Z3 and Fe-PZ highlights the complex interplay between active oxidation and acid-catalyzed side pathways. Although both systems generated comparable average methanol rates (35.6 and 36 μmol g_cat_^–1^ h^–1^, respectively), their stability profiles diverged markedly. While Fe-PZ maintained steady operation dominated by methanol-to-olefin dehydration, Fe-Z3 shifted rapidly toward a heavy, coke-dominated carbon-loss pathway (48.9% C-selectivity, Table S2), coincident with its pronounced time-on-stream deactivation. This trend parallels previous continuous Fe-zeolite/N_2_O studies in which carbon deposition was experimentally identified as a contributor to declining catalytic activity. Importantly, however, coke formation has been described as only a partial origin of deactivation in such systems, indicating that changes in the zeolite framework and/or accessible Fe-site population may contribute concurrently.^38,82^

Postreaction XRD revealed substantial changes in the crystalline structure of the catalysts (Figures 10 and S7).

**Figure 10:**
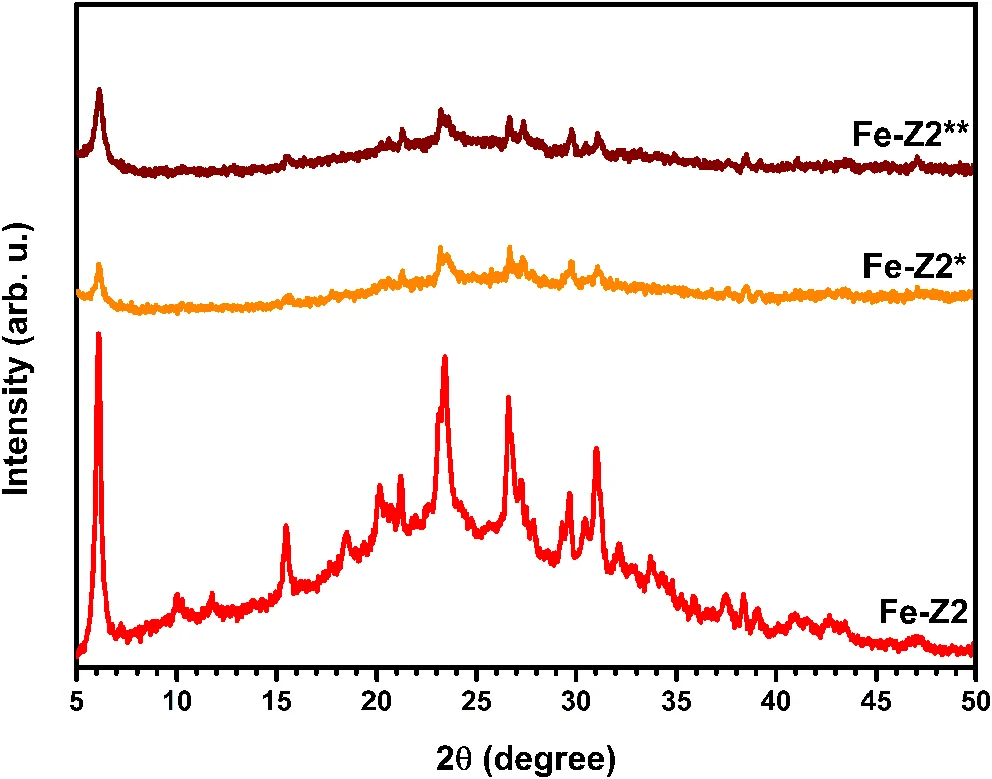
XRD patterns of fresh Fe-FAU samples of fresh (Fe-Z2), Fe-Z2 after reaction (Fe-Z2*) and Fe-Z2 after regeneration (Fe-Z2**).

For Fe-Z2, the characteristic FAU reflections were strongly attenuated after reaction and remained suppressed following regeneration, indicating that the initial long-range crystalline order was not restored after the reaction–regeneration cycle. The regenerated Fe-Z1, Fe-Z3, and Fe-PZ catalysts likewise exhibited varying degrees of residual FAU crystallinity. Similar postreaction structural changes have been reported for Fe-zeolite catalysts, where decreased zeolite crystallinity was associated with structural deformation accompanying catalytic operation and coke deposition.^83^ More broadly, characterization of fresh and used Fe-zeolites under continuous CH_4_/N_2_O conversion has shown that catalyst evolution during reaction coincides with the accumulation of retained carbonaceous species implicated in deactivation.^80^ Nonetheless, the extent of retained crystallinity in the present Fe-FAU series did not directly parallel the time-on-stream stability profiles, as Fe-Z2 maintained the most stable methanol productivity despite substantial loss of long-range order.

Collectively, these overarching reaction profiles demonstrate that source dependent matrix effects strongly influence active Fe site evolution, framework acidity moderation, and competitive surface reaction pathways. Even under identical metal-incorporation protocols, the resulting catalysts exhibit source-specific properties inherited from their parent industrial byproducts. These intrinsic features influence the final distribution of active Fe species, the boundaries of hydrothermal dealumination, and the balance between primary oxidation and secondary dehydration networks. Consequently, these findings support a matrix-guided interpretation for waste-to-catalyst approaches, wherein industrial residues are treated not as simple, passive precursors, but as inherited chemical matrices whose framework-forming components, recoverable metal fractions, and inherited cocation chemistry can be considered as interacting catalyst-design parameters.

## 4 Conclusion

This study investigated source-dependent fly ash matrix effects in Fe-FAU zeolite catalysts using methane-to-methanol conversion as a structure-sensitive diagnostic reaction. Coal fly ash was fractionated into an acid-treated aluminosilicate residue and an Fe-rich leachate, which were reintegrated through OSDA-free FAU synthesis, Fe­(II) oxalate recovery, recovered-Fe incorporation, and steaming.

The recovered Fe salt generated catalytically relevant Fe environments, including isolated Fe^3+^ descriptors associated with the highest initial methanol formation rate observed in this study, 137 μmol g_cat_^–1^ h^–1^, although Fe recovery efficiency remained source-dependent. Catalytic performance was governed by matrix-dependent redistribution of residual cocations, retained native Fe, framework Al, and recovered Fe species during crystallization, steaming, and MTM operation. In the most favorable matrix, residual alkali and alkaline-earth species lowered apparent FAU crystallinity but moderated acid-site accessibility and preserved small extra-framework Fe–oxo/hydroxo species, enabling Fe-Z2 to show more stable methanol productivity and higher methanol selectivity than the more crystalline Fe-Z3 and pure FAU-derived Fe-PZ references. Conversely, stronger dealumination or higher Fe/Ca imbalance produced less accessible extra-framework Fe species, Fe_2_O_3_-like aggregation, secondary hydrocarbon formation, and faster deactivation.

## Supplementary Material



## Notes

Deconvoluted UV–vis spectra of Fe-FAU samples (Figure S1); N_2_ adsorption–desorption isotherms of FAU and Fe-FAU samples (Figure S2); deconvoluted NH_3_-TPD spectra of Fe-FAU samples (Figure S3); NH_3_-TPD deconvolution fitting parameters, including peak centers, fwhm values, integrated areas, reduced χ^2^, and adjusted *R* ^2^ values (Table S1); XRD patterns from repeat synthesis and steaming experiments for Z2 and Fe-Z2 samples (Figure S4); coke-excluded time-on-stream selectivity evolution of detected products during methane-to-methanol conversion over Fe-FAU catalysts (Figure S5); methane-to-methanol reaction results obtained under CH_4_/N_2_O/H_2_O/He flow conditions (Table S2); representative short-time methane-to-methanol repeat tests for the Fe-Z2 route showing initial methanol productivity trends (Figure S6). The XRD patterns of regenerated Fe-FAU catalysts­(Figure S7)­(PDF) (PDF)
