# Supplementary material for: Valorization of Coal Fly Ash into Fe-FAU Catalysts: Targeted Iron Recovery and Fly Ash Matrix Effects in Methane-to-Methanol Conversion
Source: ACS Omega. 2026 Oct 24;11(44):69038–53. doi: 10.1021/acsomega.6c09000 (PMC13756665; doi:10.1021/acsomega.6c09000)
Supplement: Supplementary file 1 [file ao6c09000_si_001.pdf]

**Supplementary Information**

# Valorization of Coal Fly Ash into Fe-FAU Catalysts: Targeted Iron Recovery and Fly Ash Matrix Effects in Methane-to-Methanol Conversion

Süleyman Şener Akın[a], Selin Cansu Gölboylu[a,c], İlhan Duruk[b],Can Barkın Dericioğlu[b], Bahar İpek[b], Burcu Akata[a,c ,*]

[a]Micro and Nanotechnology Department, Middle East Technical University, Ankara,06800, Turkey

[b] Chemical Engineering Department, Middle East Technical University, Ankara, 06800, Turkey

[c] Central Laboratory, Middle East Technical University, Ankara 06800, Turkey

**Section 1: Deconvoluted of UV-VIS and NH3-TPD Spectra and BET isotherms of Fe-FAU samples in this study**

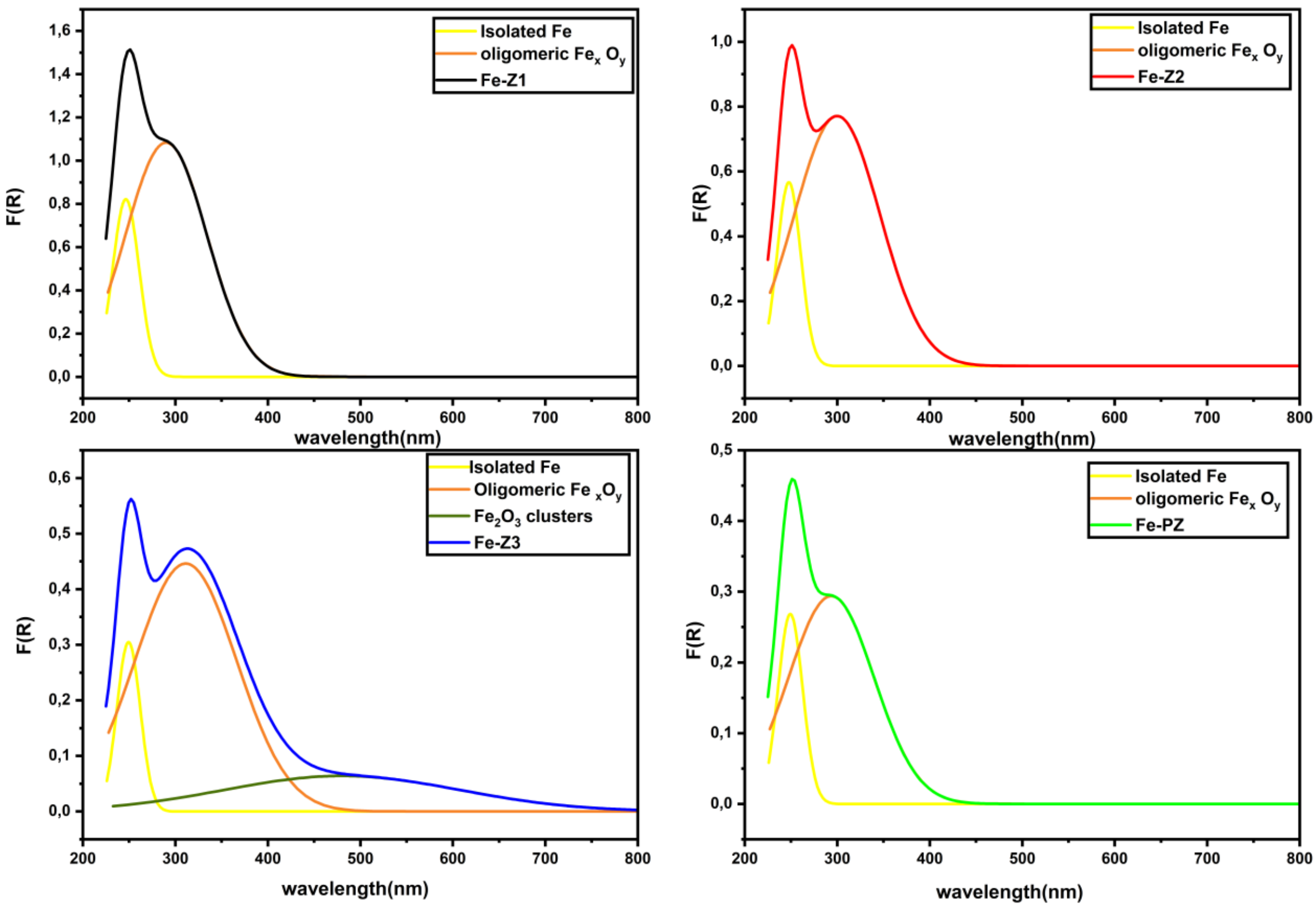


**Figure S1.** Deconvoluted UV-VIS Spectra of Fe-FAU samples

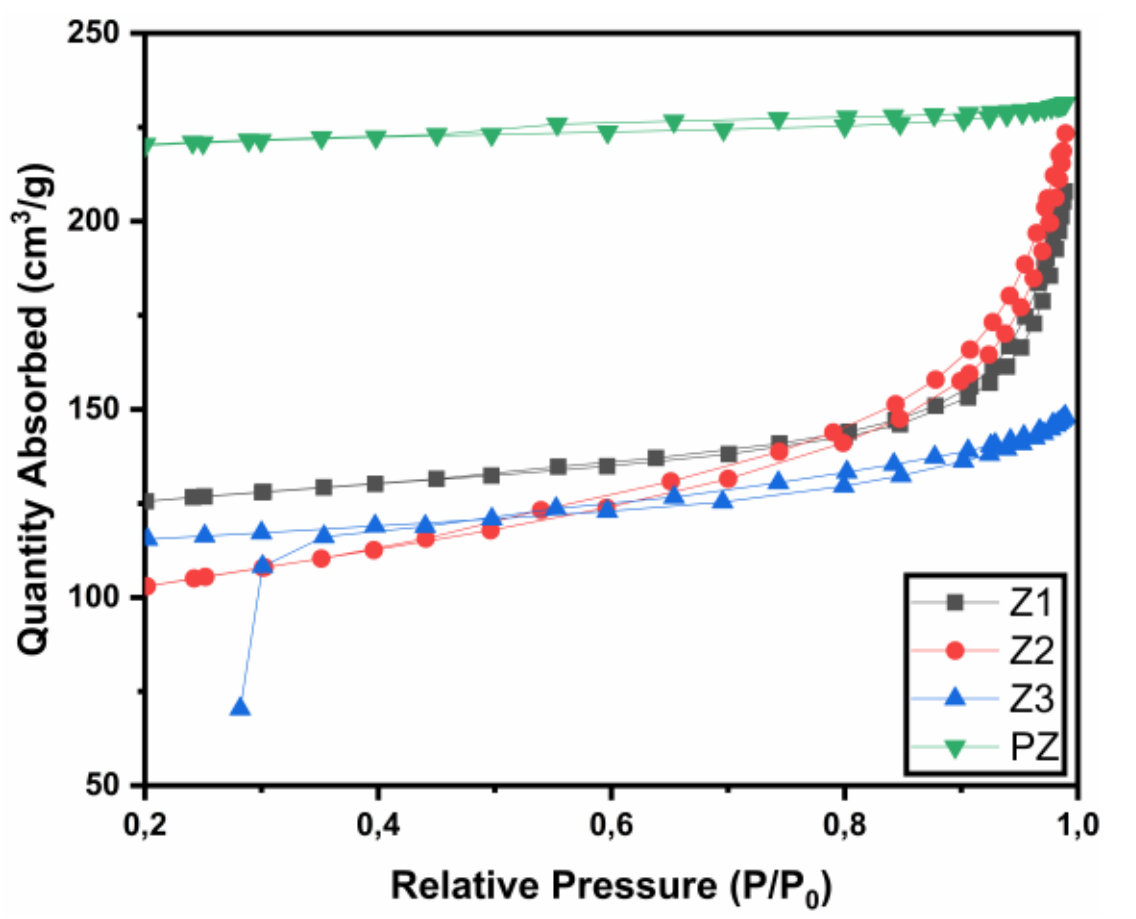


(a) BET Isotherms of synthesized FAU samples in this study

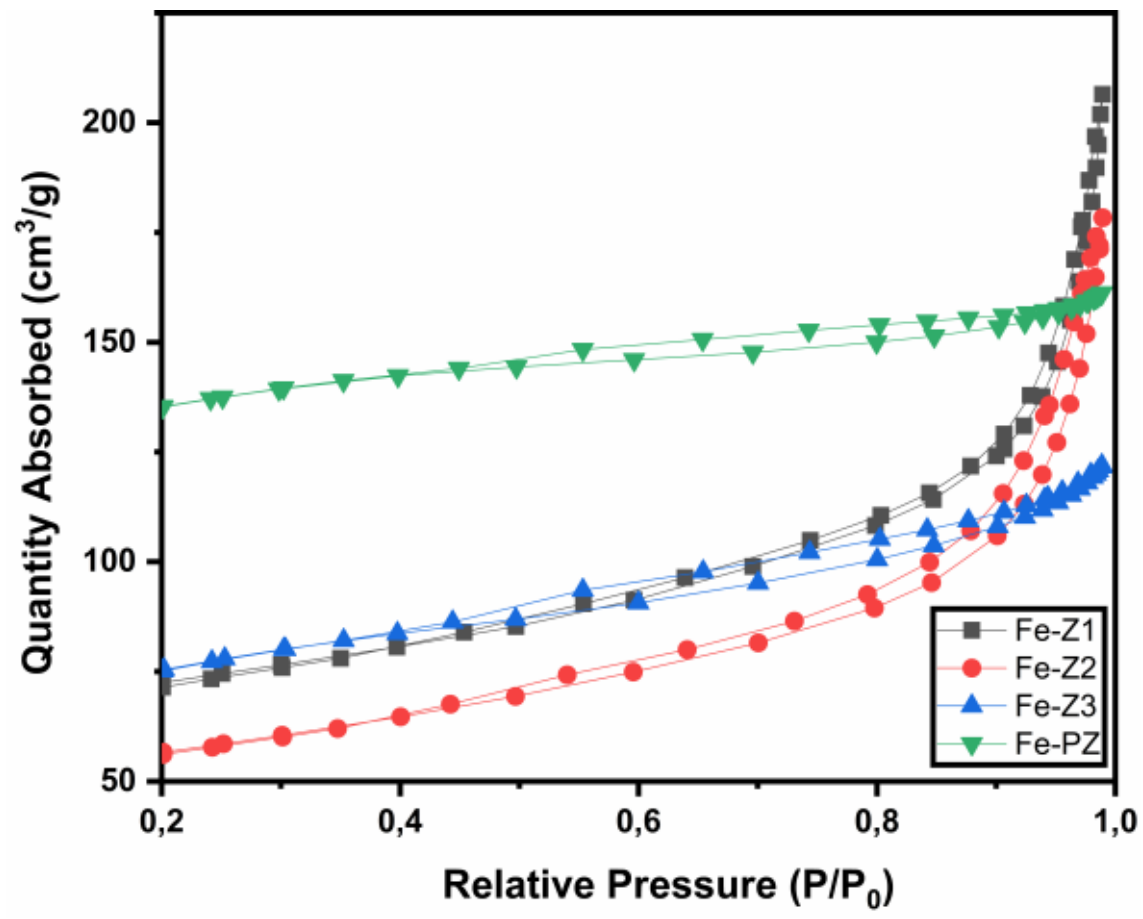


(b) BET Isotherms of Fe- FAU samples in this study

**Figure S2.** BET Isotherms of (a) FAU samples (b) Fe-FAU samples in this study

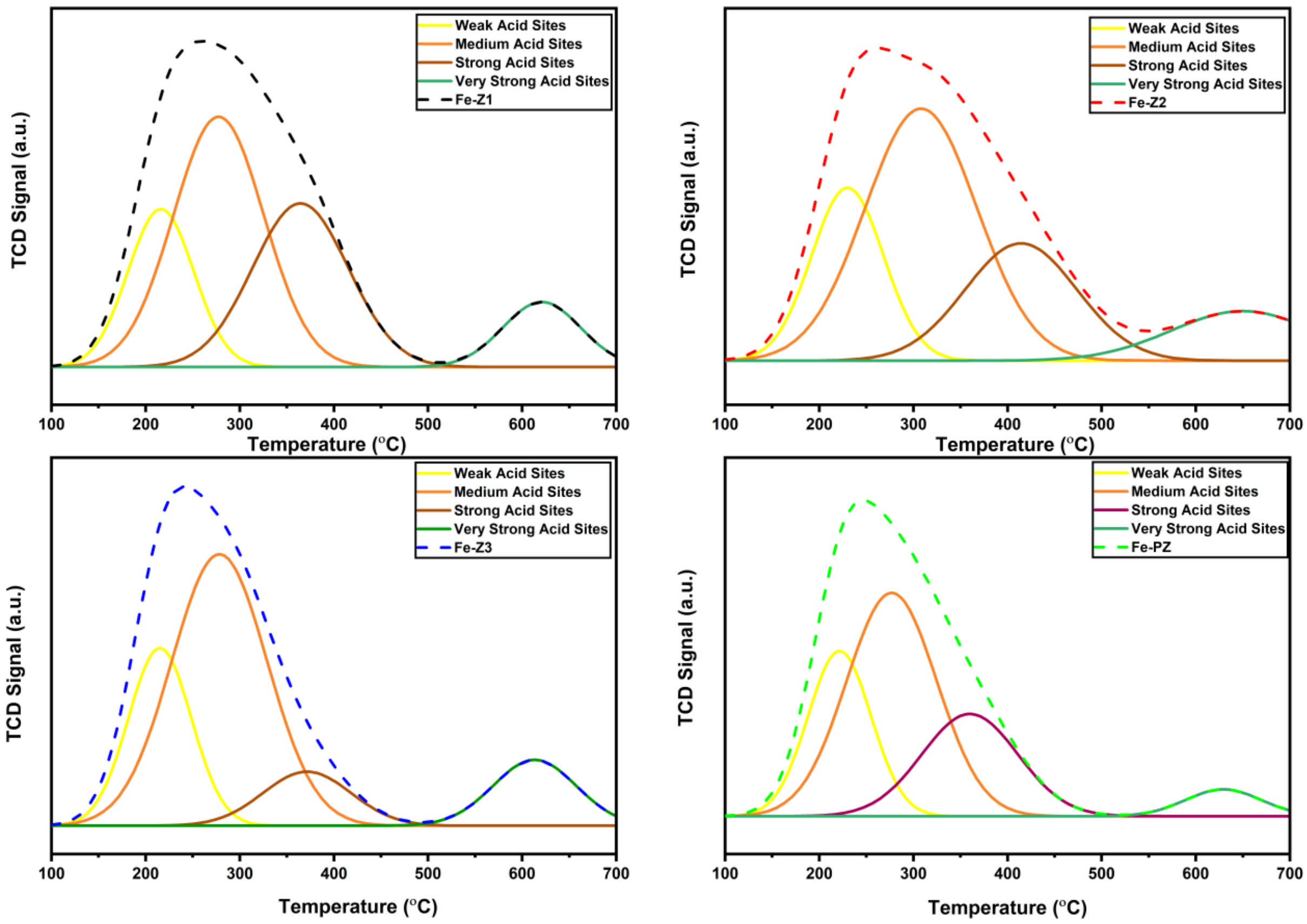


**Figure S3.** Deconvoluted $NH_3$-TPD Spectra of Fe-FAU samples

**Table S1.** $NH_3$-TPD deconvolution fitting parameters of Fe-FAU catalysts. Peak centers, FWHM values, and integrated areas correspond to the fitted components shown in Figure S3. Reduced $\chi^2$ and adjusted $R^2$ values correspond to the overall fit quality for each sample.

| Sample | Component | Peak center, $T_{max}$ (°C) | FWHM (°C) | Integrated area | Reduced $\chi^2$ | Adjusted $R^2$ |
|---|---|---|---|---|---|---|
| **Fe-Z1** | Peak 1 | 216 | 82 | 0,73 | $1{,}7*10^{-8}$ | 0,99 |
| | Peak 2 | 278 | 113 | 1,60 | | |
| | Peak 3 | 364 | 120 | 1,11 | | |
| | Peak 4 | 621 | 97 | 0,35 | | |
| **Fe-Z2** | Peak 1 | 230 | 90 | 0,75 | $4{,}3*10^{-8}$ | 0,99 |
| | Peak 2 | 308 | 140 | 1,70 | | |
| | Peak 3 | 414 | 140 | 0,79 | | |
| | Peak 4 | 650 | 180 | 0,33 | | |

| Sample | Component | Peak center, $T_{max}$ (°C) | FWHM (°C) | Integrated area | Reduced $\chi^2$ | Adjusted $R^2$ |
|---|---|---|---|---|---|---|
| **Fe-Z3** | Peak 1 | 215 | 79 | 0,53 | $1,1*10^{-8}$ | 0,99 |
| | Peak 2 | 278 | 119 | 1,22 | | |
| | Peak 3 | 371 | 110 | 0,22 | | |
| | Peak 4 | 613 | 104 | 0,25 | | |
| **Fe-PZ** | Peak 1 | 222 | 79 | 0,74 | $1,06*10^{-8}$ | 0,99 |
| | Peak 2 | 277 | 113 | 1,42 | | |
| | Peak 3 | 360 | 120 | 0,69 | | |
| | Peak 4 | 629 | 95 | 0,14 | | |

*Integrated areas were converted to $NH_3$ amounts using the calibration factor of 0,213 obtained from the $NH_3$-TPD calibration procedure. The calculated acidity values are reported in Table 6.

**Section 2: Representative Repeat Experiments for the Fe-Z2 Route**

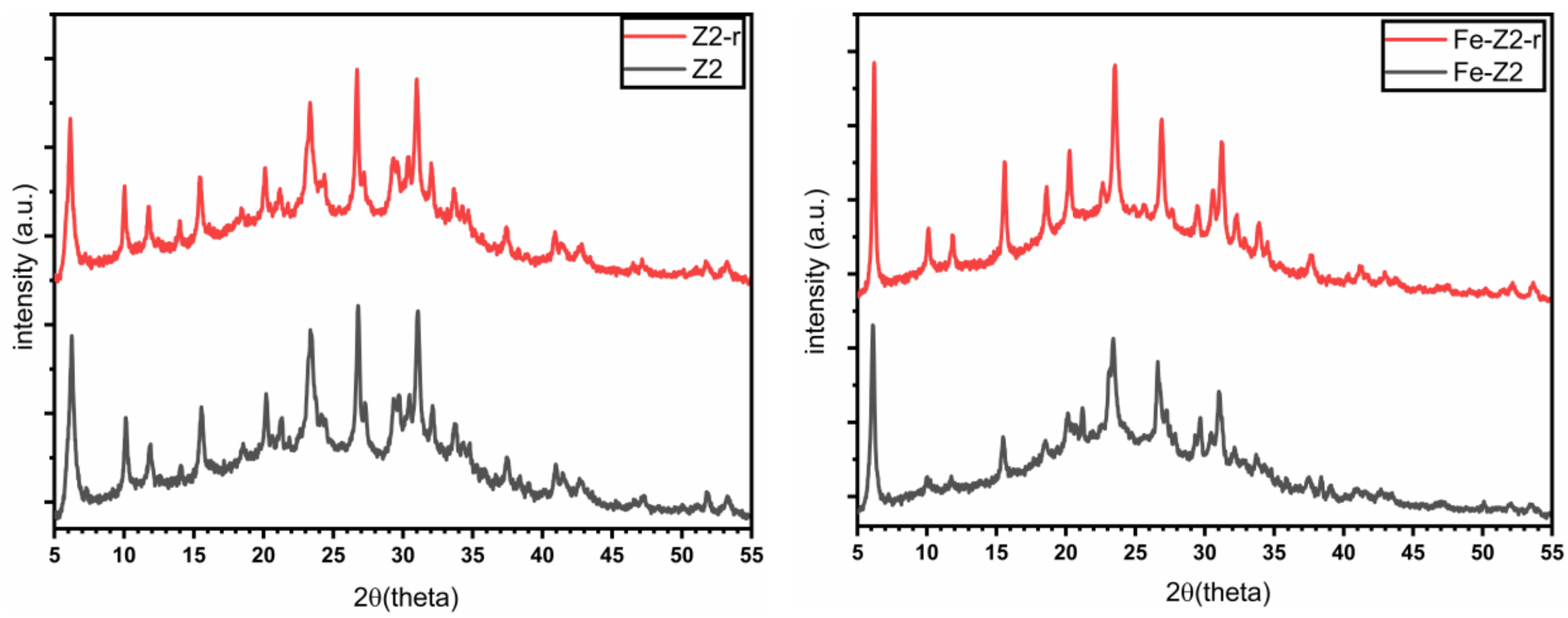


**(a)XRD Patterns of Z2 samples** **(b) XRD Patterns of Fe-Z2 Samples**

**Figure S4.** XRD patterns of repeat synthesis and steaming experiments for (a) Z2 and (b) Fe-Z2 samples.

## Section 3: Methanol Formation Activity

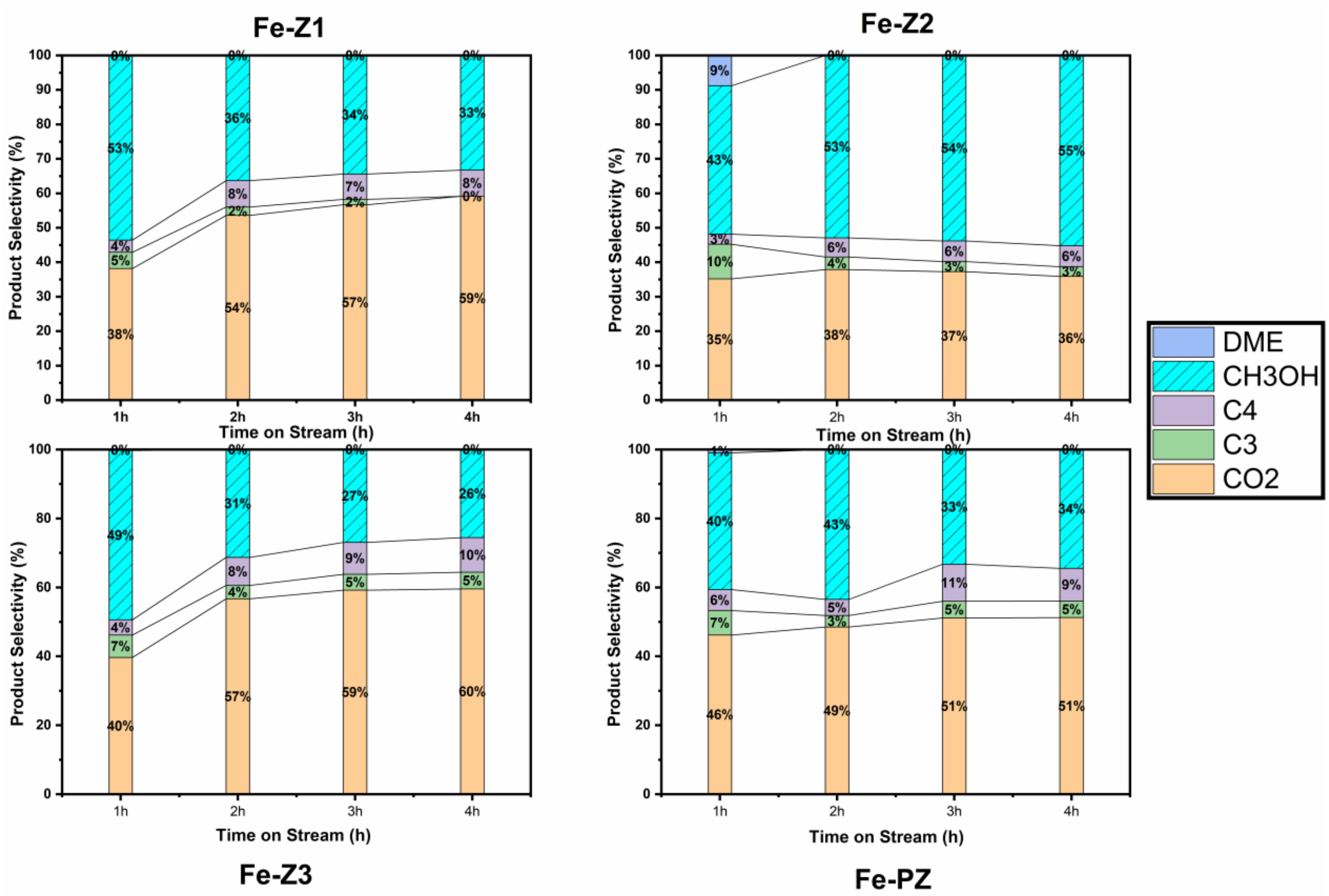


**Figure S5.** Coke-excluded time-on-stream selectivity evolution of detected reaction products during the MTM reaction over Fe-FAU catalysts.

**Table S2.** Methane conversion to methanol reaction results obtained at 300 °C and 30,4 kPa CH4, 10,12 kPa N2O, 9 kPa $H_2O$, balance He (~100 mg catalyst, 100 sccm total flow, GHSV= 60000 ml g-1 h-1)

| | Catalyst | | | |
|---|---|---|---|---|
| | **Fe-Z1** | **Fe-Z2** | **Fe-Z3** | **Fe-PZ** |
| **$CH_4$ Conversion/%** | 0,03 | 0,06 | 0,03 | 0,02 |
| **$S_{MeOH}$** | 16(33,2)* | 25(41,2)* | 14(27,4) | 16(30)* |
| **$S_{MeOH+DME}$** | 16(33,3)* | 28,4(45,4)* | 14,1(27,6)* | 16,2(30,3)* |
| **$S_{CO+CO2}$** | 18,5(38,5)* | 18,6(29,8)* | 20,5(40,1)* | 19,8(37,1)* |
| **$S_{C2-C4}$** | 13,5(28,2)* | 17,1(27,3)* | 15,7(30,7)* | 15,9(29,7)* |
| **$S_{Coke}$** | 52,0 | 37,5 | 48,9 | 46,5 |

* Carbon-based selectivity excluding coke formation.

**Section 4: Representative Repeated MTM tests for Fe-Z2 sample**

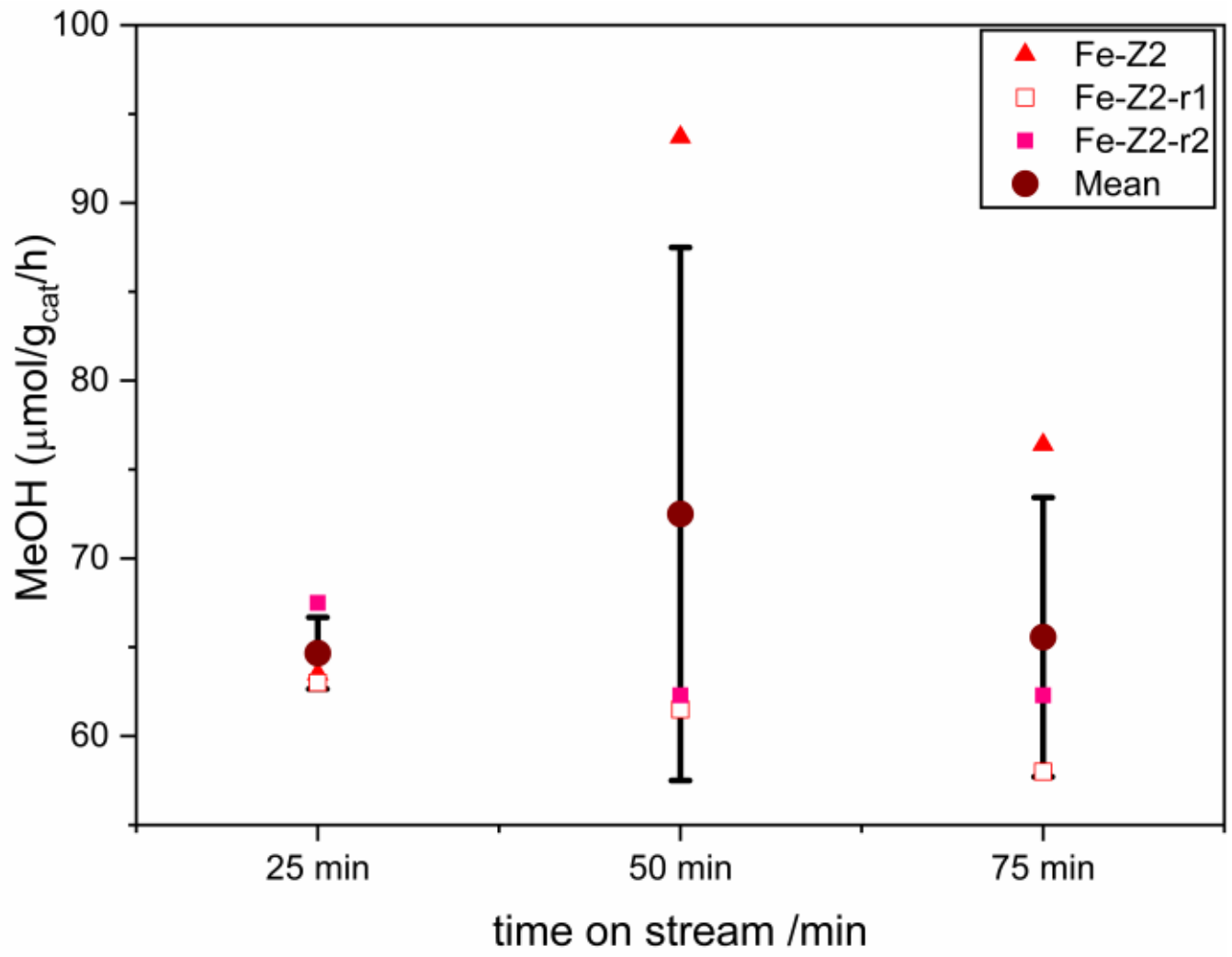


**Figure S6.** Representative short-time MTM repeat tests for the Fe-Z2 route, showing methanol productivity over the initial reaction period. The data are presented to compare the short-time methanol productivity trend among repeated Fe-Z2 tests.

**Section 5: XRD Analysis of Regenerated Fe-FAU catalyst samples**

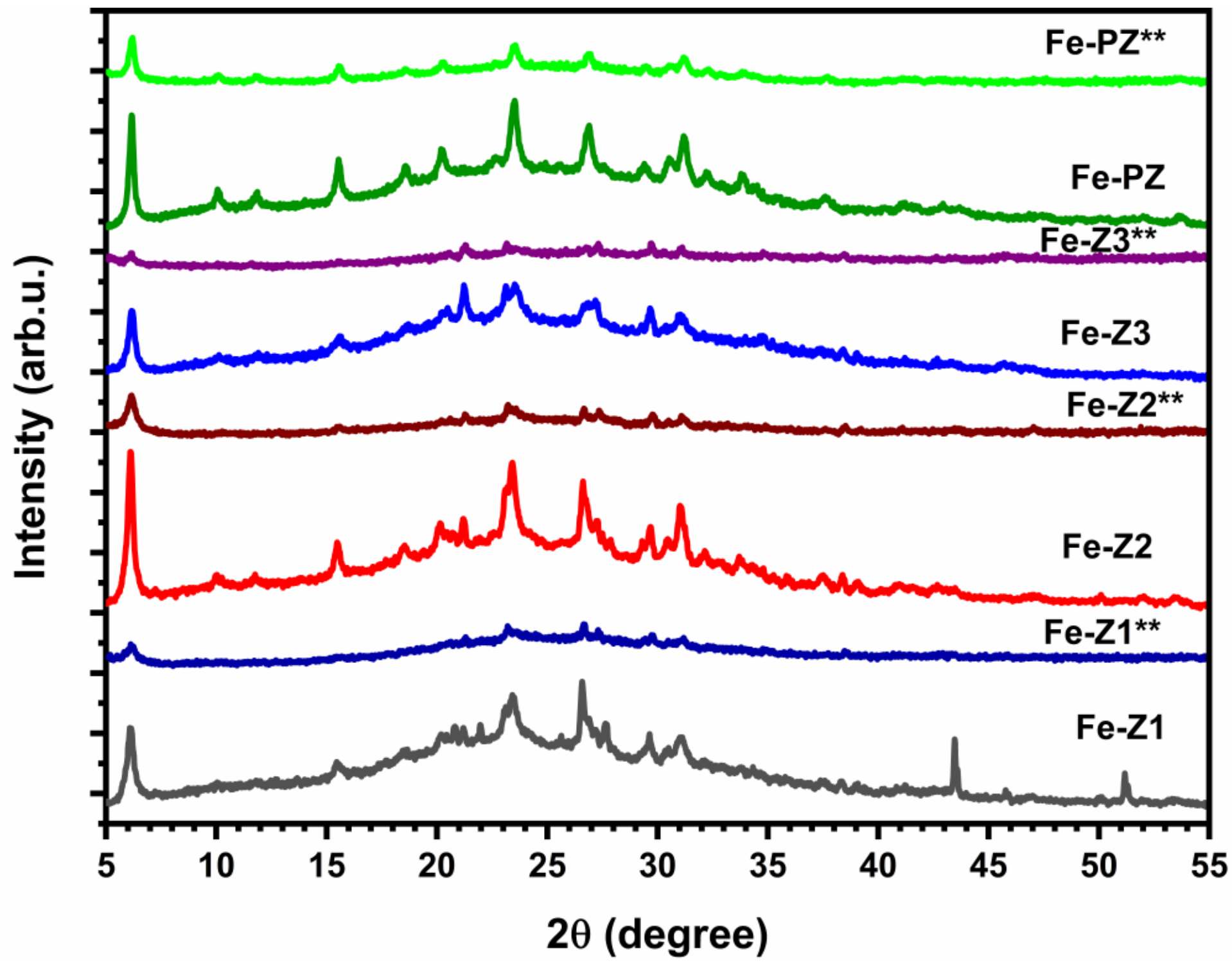


**Figure S7.** XRD patterns of regenerated Fe-FAU catalyst samples. ** represents regenerated samples of fresh catalyst.

**Section 6: GC Analysis Details**

Table S3: Details of the used methods

| | Method 1 | Method 2 |
|---|---|---|
| Used Columns | HP-Plot-Q + CP Molecular Sieve 5A | HP-Plot-Q |
| Injection Temperature/ °C | 150 | 150 |
| Split | Splitess | 5:1 |
| TCD Temperature/ °C | 250 | 250 |
| FID Temperature/ °C, $H_2$ flow & Air flow / ml/min | 300, 60 & 300 | 300, 40 & 750 |
| Column flow pressure / psi | 21.37 | 13.56 |
| Oven method | 40 °C for 5 min, 160 °C for 10 min, 10 °C/min ramp | 50 °C for 8 min, 160 °C for 8 min, 20 °C/min ramp |
